# Angiogenesis in multiple sclerosis and experimental autoimmune encephalomyelitis

**DOI:** 10.1186/s40478-014-0084-z

**Published:** 2014-07-22

**Authors:** Francesco Girolamo, Cristiana Coppola, Domenico Ribatti, Maria Trojano

**Affiliations:** Department of Basic Medical Sciences, Neurosciences and Sense Organs, University of Bari ‘Aldo Moro’, Piazza Giulio Cesare, 11, 70124 Bari, Italy; National Cancer Institute ‘Giovanni Paolo II’, via O. Flacco, 65, 70124 Bari, Italy

**Keywords:** Angiogenesis, Blood–Brain Barrier, Experimental Autoimmune Encephalomyelitis, Multiple Sclerosis, Neuroprotection, Neurovascular uncoupling, Neurovascular unit, Vascular Endothelial Growth Factor

## Abstract

Angiogenesis, the formation of new vessels, is found in Multiple Sclerosis (MS) demyelinating lesions following Vascular Endothelial Growth Factor (VEGF) release and the production of several other angiogenic molecules. The increased energy demand of inflammatory cuffs and damaged neural cells explains the strong angiogenic response in plaques and surrounding white matter. An angiogenic response has also been documented in an experimental model of MS, experimental allergic encephalomyelitis (EAE), where blood–brain barrier disruption and vascular remodelling appeared in a pre-symptomatic disease phase. In both MS and EAE, VEGF acts as a pro-inflammatory factor in the early phase but its reduced responsivity in the late phase can disrupt neuroregenerative attempts, since VEGF naturally enhances neuron resistance to injury and regulates neural progenitor proliferation, migration, differentiation and oligodendrocyte precursor cell (OPC) survival and migration to demyelinated lesions. Angiogenesis, neurogenesis and oligodendroglia maturation are closely intertwined in the neurovascular niches of the subventricular zone, one of the preferential locations of inflammatory lesions in MS, and in all the other temporary vascular niches where the mutual fostering of angiogenesis and OPC maturation occurs. Angiogenesis, induced either by CNS inflammation or by hypoxic stimuli related to neurovascular uncoupling, appears to be ineffective in chronic MS due to a counterbalancing effect of vasoconstrictive mechanisms determined by the reduced axonal activity, astrocyte dysfunction, microglia secretion of free radical species and mitochondrial abnormalities. Thus, angiogenesis, that supplies several trophic factors, should be promoted in therapeutic neuroregeneration efforts to combat the progressive, degenerative phase of MS.

## Angiogenesis in MS

Multiple Sclerosis (MS) is an autoimmune demyelinating disease of the Central Nervous System (CNS) whose cause remains elusive. An inappropriate recognition of an autoantigen on myelinated nerve fibers recruits macrophages and lymphocytes in the CNS, leading to white and grey matter demyelination. Other pathological hallmarks of the disease are gliosis, axon degeneration and remyelination attempts.

An altered Blood–Brain Barrier (BBB) permeability, with a subsequent transmigration of lymphocytes and mediators into the CNS, is an early event in the MS pathogenesis. Local breakdown of BBB has been demonstrated, as gadolinium-DTPA enhancement (gd.e.) on T1 weighted magnetic resonance imaging (MRI), to precede other clinical signs and to be a prominent event in disease progression [1,2]. BBB incompetence has also been documented as an altered expression of endothelial tight junction proteins, changes of vascular basement membrane (BM) molecules and pericytes in acute and progressive MS forms [3–10]. Localized BBB disruption could precede the development of typical demyelinating lesions associated with inflammatory cuffs around veins or venules [1,2,11,12]. However, macrophage infiltration also seems possible across a preserved BBB for humoral factors (marked by the absence of gd.e.), as already demonstrated in MS [13,14] and EAE [15]. The increased BBB permeability is primarily, but not only, driven by the release of Vascular Endothelial Growth Factor (VEGF)/vascular permeability factor [16], that also regulates vessel growth and is chemotactic for monocytes and lymphocytes, promoting neuroinflammation [17–19]. Other BBB permeability promoting factors such as interferon-γ (IFN-γ), tumour necrosis factor-α (TNF-α) and interleukin-1 (IL-1) have been described in MS [reviewed in 7] (Table 1).

**Table 1:** **Reported angiogenesis-related changes in serum, CSF and PBMCs of MS patients**

**Angiogenesis related molecules**	**Serum**		**CSF**		**PBMCs**	
VEGF-A	**↑**	[12,59,255]	**↓**	[53,63]	**↓**	[59,63]
VEGF-D	**↑**	[256]				
VEGF-R3	**↑**	[256]				
Angiopoietin-2	**↑**	[256]				
Basic FGF	**↑**	[12]	**↑**	[257]		
Endothelin-1	**↑**	[85,184]	**↑**	[185]	**↑**	[258]
Nitric oxide and NOS	**↑**	[259]	**↑**	[111,185]	**↑**	[260]
TNF-α	**↑**	[261]	**↑**	[258,262,263]	**↑**	[264–266]
TGF-β	**↑**	[267]	**↑**	[268]	**↓**	[269]
	**↓**	[268]				
IFN-γ	**↑**	[270]	**↑**	[75]	**↑**	[265,269]
MMP-2	**↑**	[271]	**↑**	[272]	**↑**	[273]
MMP-9	**↑**	[271,274]	**↑**	[275,276]	**↑**	[273]
TIMP-1	**↑**	[274]	**↓**	[277]	**↓**	[278]
sCD146			**↑**	[279]		

So-called sprouting angiogenesis, the formation of new vessels from pre-existing ones, is a strictly controlled process during tissue repair and regeneration to provide the necessary oxygen and nutrients for an area with increased cellular needs. Angiogenesis is a vital process in growth and organ development; it is active in developing human CNS [20] but quiescent in adult human brain [21]. Endothelial cell proliferation and a consistent increase of vascular networks due to angiogenesis have been investigated and found in MS demyelinating lesions only by two groups [22,23] and much remains to be demonstrated about the regulation and significance of angiogenesis in MS.

Increased angiogenesis is a common feature of several neurological conditions, with detrimental effects as observed in Alzheimer’s disease (AD) [24], Parkinson’s disease (PD) [25] and brain tumours [26], whereas a beneficial effect of angiogenesis has been proposed in cerebrovascular ischemic and traumatic brain injury [27]. In MS lesions and in surrounding normal-appearing white matter (NAWM) and grey matter (NAGM), an angiogenic response has been suggested to contribute to disease progression [28] or, alternatively, to remission after relapses.

Ever since the first descriptions of MS disease signs, the vascular component has been acknowledged as an important element to understand the disease pathogenesis [29–33]; breakdown of the BBB in MS lesions was first described by Broman [34]. Acute and chronic demyelinating lesions and even NAWM of MS patients show blood vessels with a glomeruloid morphology [35], class II MHC antigen expression, intramural fibrin, hemosiderin, and collagen deposition, vessel wall hyalinization, evidence of thrombi and haemorrhages and iron accumulation [36], all features consistent with angiogenesis and endothelial cell proliferation [23,37].

One explanation of the angiogenic response seen in NAWM may be an effect of the increased energy demand for impulse conduction along excitable demyelinated axons, together with a reduced axonal ATP production due to mitochondrial dysfunction, both inducing a chronic state of ‘virtual’ hypoxia in chronically demyelinated axons [38]. Meanwhile, chronic inflammation itself is pro-angiogenic and, in turn, VEGF is a pro-inflammatory factor.

## Angiogenesis in EAE

**Figure 1:**
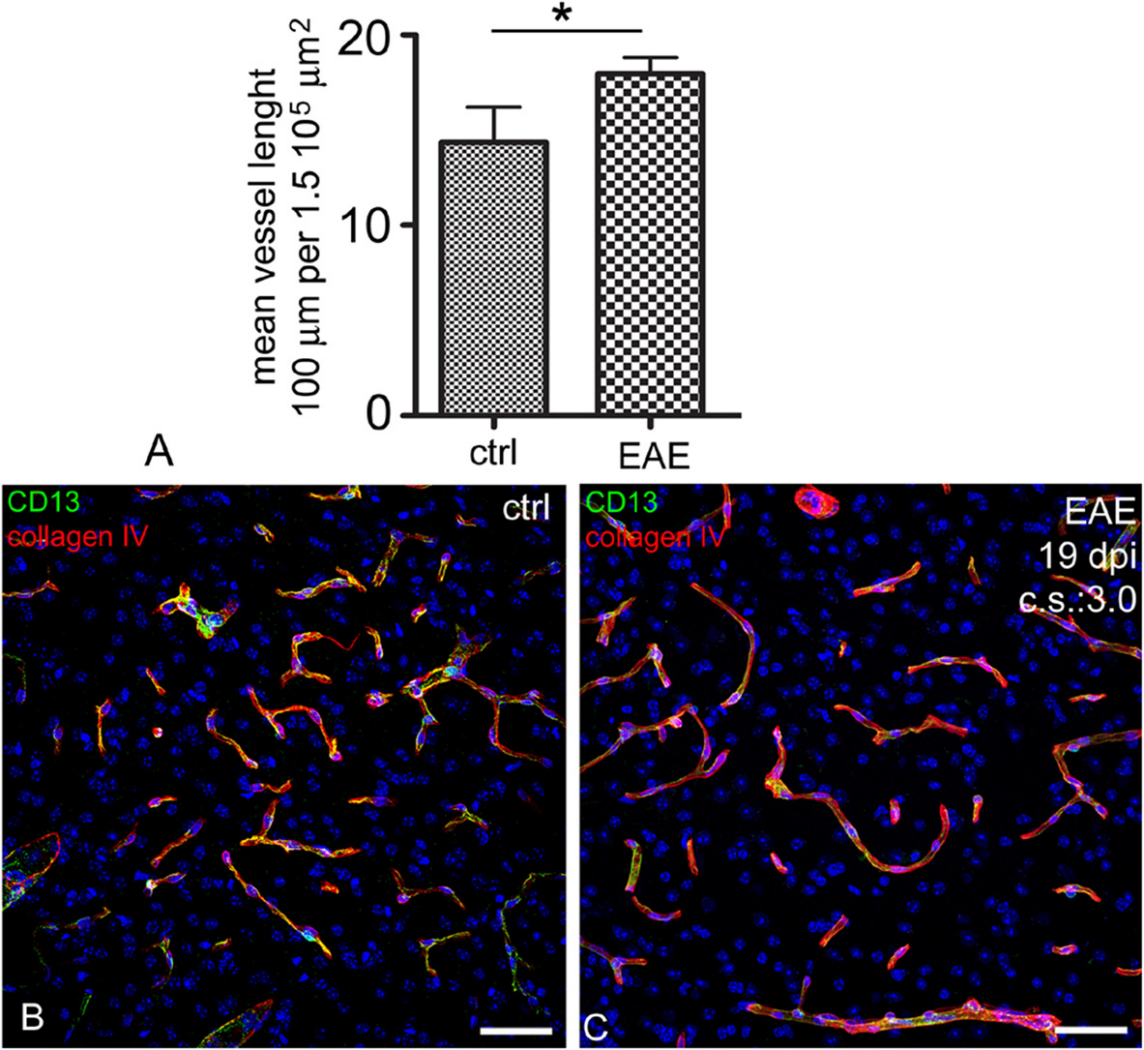
**Vessel density is increased in EAE mice. A**. Vessel density, calculated as the cumulative vessel profile length per standard area (ImageJ software, NIH, Bethesda, USA; observer blinded to section ranking: CC), is significantly increased in EAE brains at 19 days post-immunization (dpi) (ctrl: 12 week-old control mice, n = 5; EAE: 12 week-old EAE mice, n = 5; t-test, p = 0.0026). **B**, **C**. Representative images of the vasculature of the same cerebral cortex area (area frontalis) in healthy mice and EAE (mouse at 19 dpi after 200 μg of MOG_35–55_ immunization; clinical onset: 10 dpi, clinical score (cs) at 19 dpi = 3.0) are immunolabelled with CD13, a pericyte marker, and collagen IV of the vessel basal lamina. Some glomeruloid microvessels associated with a high number of pericytes are visible in the EAE brains.

A good animal model for MS is experimental allergic encephalomyelitis (EAE). It can be induced by immunization using antigens derived from myelin. These antigens elicit an acute demyelinating process driven by T cells and macrophages which can have a chronic relapsing course quite similar to MS. Several reports indicate early BBB breakdown in the CNS of EAE [39–44]. Increased vessel density has been documented in different experimental models, including EAE induced in the mouse [40,45,46], guinea pig [47–49], and Lewis rat during the relapse phase [50]. Figure 1 shows our results on EAE induced by MOG_(35–55)_ immunization in C57Bl/6 J cerebral cortex vasculature, demonstrating an increased angiogenesis (cumulative vessel length) as compared to control mice.

Boroujerdi and co-worker [40] demonstrated that vascular remodelling is an early process in MOG-induced EAE, because increased vessel areas and endothelial proliferation appeared evident as early as 4 days post-immunization (dpi), in a pre-symptomatic disease phase, while the onset of symptoms occurred around 14 dpi. In the EAE, VEGF is expressed by astrocytes, monocytes and activated Th1 lymphocytes, all contributing to BBB breakdown [35,39,51]. Other studies have confirmed increased angiogenesis, severe inflammation and activated VEGF signalling in inflamed lesions [35,46,47,50,52]. VEGF increases inflammation in those areas injected with exogenous VEGF in MBP-immunized animals [35]. In addition, the expression of VEGF is demonstrated in dorsal root ganglion neurons and dorsal column axons in spinal cord, implying that it may act as a neuromodulator [45]. During EAE, an increased neuronal VEGF expression has been described in the early phase but decreased expression in the late phase [45]. Another study reported a decreased VEGF level not only in neurons but also in astroglia in a rat EAE experimental model [53]. A VEGF decrease may be caused by neuronal dysfunction, as already demonstrated in epilepsy by McCloskey et al. [54]. Astroglial production of VEGF is enhanced in pathological conditions, including human astrocytoma [55] and MS and EAE, to promote angiogenesis and glial survival [50]. The striking differences of VEGF expression levels and cell sources among different studies could be explained by the use of different EAE models as regards immunization protocol, animal species and strain employed.

## The role of VEGF in MS pathogenesis

Accumulating evidence indicates a role for VEGF in the pathogenesis of MS. VEGF-A, mainly secreted by astrocytes and neurons but also by cerebral endothelial cells and leukocytes, binds its receptors, VEGF-R1 and VEGF-R2, expressed on different cell types including endothelial cells, astrocytes, neurons, microglia, leukocytes [19,39,45,47,50,53,56,57]. An elevated VEGF expression was detected in reactive astrocytes of both active and inactive chronic demyelinated lesions [35], in NAWM from post mortem MS brains [58], and in sera of MS patients during clinical disease relapses [59], and is correlated with the length of spinal cord lesions [12].

VEGF, acting as a pro-inflammatory factor, can cause CNS injury. The effect of VEGF in other disease models could also shed light on the MS pathogenesis. In an ischemia-reperfusion model, inhibiting the activity of endogenous VEGF reduces the size of lesions [60], whereas exogenous administration of VEGF exacerbates CNS injury [35,57,61]. However, in experimental conditions, the administration of VEGF to the CNS can be beneficial or detrimental depending on the rat strain, VEGF dose and, especially, timing [61,62].

In the late MS phase, VEGF-A, acting as a neuroprotective agent for neurons and neural progenitors, is decreased in the cerebrospinal fluid (CSF) of MS patients and also in peripheral blood mononuclear cells (PBMC) from secondary progressive MS (SP-MS) patients [53,63]. In addition, reduced levels of VEGF are associated with EAE, as already mentioned [45,53,64], and also with amyotrophic lateral sclerosis (ALS), a human neurodegenerative disease [65–67].

VEGF is released for neuroprotection purposes, enhances axonal growth and neuronal resistance to injury of cultured neurons [68], but at the same time it induces the dismantling of BBB tight junctions [69].

VEGF-A is mitogenic for astrocytes [70], and reactive astrocytes play a pivotal role in the healing process after spinal cord injury [71]. VEGF-R1 and R2 are upregulated on microglia and other antigen presenting cells after CNS trauma, suggesting a modulating role of VEGF in CNS immune surveillance [72].

VEGF induces anti-inflammatory effects and downregulation of a broad set of inflammatory cytokines and chemokines in microglia/macrophages, and this immunosuppression is linked to the plasticity-promoting action of VEGF [73]. But VEGF-A also recruits monocytes via VEGF-R1, inducing inflammation and BBB breakdown in rat brain [19], as well as being chemotactic for T-cells and macrophages [74]. T cells express VEGF and VEGF-R2, fostering the transition toward the Th1 phenotype; an upregulation of Th1 lymphocytes in CSF has been observed in relapsing MS patients [75]. VEGF-stimulated T cells also exacerbated adoptive EAE in rats [51].

VEGF production is promoted by several pro-inflammatory cytokines such as IL-1β, IL-1α and IL-18 [76], and macrophages, too, are known to produce VEGF [27] and NO, further stimulating VEGF production and VEGF receptor expression by endothelial cells [36].

VEGF-R2 is also expressed on endothelial cells in active MS lesions [50], possibly contributing to produce an increased vessel density and endothelial proliferation. In response to VEGF, activated endothelial cells down-regulate Cx43 gap junctional communication [77] and increase the expression of cell adhesion molecules such as PECAM-1/CD31, VCAM, ICAM-1, MIP-1α, MHC I and II, Selectin [19], activating a loop that boosts neuroinflammation and angiogenesis. Thus, the surprisingly multiple effects of VEGF in CNS can be reconciled, considering that VEGF splice variants could result in opposite effects due either to binding with different affinity VEGF-Rs and Neuropilin-1 or to differential tyrosine residue phosphorylation of VEGFRs [78,79].

## Other angiogenic molecules potentially involved in MS and EAE angiogenesis

Hypoxia inducible factor (HIF-1α) dimerizes with HIF-1β and the complex translocation to the nucleus promotes VEGF transcription [80]. The VEGF-A gene contains a hypoxia responsive element that binds HIF-1α [80]. An increased expression of HIF-1α was demonstrated in MS lesions showing histopathological features of hypoxic tissue damage [81]. HIF-1α is also increased in EAE mice, together with other genes involved in cell migration across the BBB [46].

Platelet-derived growth factor (PDGF) and basic fibroblast growth factor (bFGF) contribute to angiogenesis [82] and oligodendrocyte progenitor growth and differentiation after demyelination [83]. Serum levels of bFGF were significantly increased in MS patients, while PDGF showed no significant change [12].

Inflammatory molecules found in MS, including IFN-γ and TNF-α, are also pro-angiogenic factors [84]. Endothelin-1 (ET-1) is another pro-angiogenic factor that is significantly elevated in MS patients [85], and antagonizing the ET-1 receptor ameliorates acute EAE [86]. Angiopoietin-2 (Ang-2) is increased in neurons, glia and inflammatory cells during EAE [45,64]. Endothelial α5β1 integrin, involved in endothelial proliferation in hypoxic conditions [87], is transiently upregulated in EAE [40]. Gene expression analysis of the laser-captured microvascular compartment of active lesions from MS autopsy samples has shown an increased expression of matrix metalloprotease-14 (MMP-14), MMP-2, ADAM17, VEGF-A, and VEGF-R1 [88]. Other inflammatory mediators such as TNF-α, IL-8, transforming growth factor-β (TGF-β), and MMP-9 released by immune cells induce angiogenesis [51] and, in turn, new vessel walls are easily permeable to immune cell transmigration and foster adhesion and cytokine molecules expression [89].

## Overlapping signalling mechanisms among angiogenesis and neurogenesis, plasticity and repair

Compelling evidence shows a coordinated interaction between the nervous and the vascular systems during development and in adult brain [90]. This interaction is responsible for the creation of a specialized perivascular microenviroment called the neurovascular niche, in which neural and glial progenitors develop, proliferate and differentiate. Adult neurogenesis primarily occurs in the subgranular zone (SGZ) in the hippocampus and the subventricular zone (SVZ) of the anterior horn of lateral ventricles. During regeneration, as well as during development, angiogenesis and neurogenesis are closely related; the molecular mediators of neurogenesis and angiogenesis overlap and cell-cell signalling between brain endothelium and neural precursors sustains ongoing angiogenesis and neurogenesis [91,92]. This crosstalk is mediated by soluble signals secreted mainly by endothelial cells [93,94]. These molecules, affecting both neural and vascular function, have been called ‘angioneurins’ [95], classified as angiogenic molecules, morphogens and growth factors; in the latter group the prototypical factor is VEGF. Endogenous VEGF, abundantly secreted by the ventricular neuroepithelium, regulates neural progenitor proliferation, migration, differentiation and the composition of neurons [96]. In adulthood, VEGF signals transmitted by VEGF-R2 and R3 enhance cell proliferation in the SVZ and SGZ by induction after voluntary motor activity [97]. Several findings implicate VEGF as a neuronal survival factor via VEGF-R1 signalling [98], and also a factor promoting oligodendrocyte precursor cell (OPC) survival and migration during axon guidance, thanks to VEGF-R2 and R3 expression [99]. Both angiogenic and neurogenic responses to VEGF are attenuated in the aged mouse brain [100]. Finally, VEGF may impact neuro-vascular interactions through alterations of the extracellular matrix molecule (ECM) composition, particularly of integrins and their ligands [101] and of SDF1/CXCR4 expression [102]. This ligand/receptor interaction is critically involved in OPC differentiation and remyelination in a model of toxic demyelination [103]. The ECM of vascular endothelial cells can trap FGF-2 (bFGF), which facilitates neurogenesis [104] and promotes OPC migration to demyelinated lesions [105]. Epidermal growth factor (EGF), pigment epithelium-derived factor (PEDF) and TGF-α have been implicated in adult neurogenesis and oligodendrogenesis [95,106]. EGF and FGF receptors co-activation is required for the maintenance of neural stem cells (NSCs) and progenitor cells in the adult SVZ [107,108]. However, prolonged exposure to EGF induces oxidative neuronal death and astrocyte commitment from NSCs [109] and a higher secretion of EGF has been demonstrated in PBMCs of patients with relapsing remitting MS (RR-MS) [110]. Neurotrophins such as nerve growth factor (NGF) and brain-derived neurotrophic factor (BDNF) reciprocally promote angiogenesis [111,112], and higher amounts of both have been detected in CSF from MS patients [113,114]. BDNF and its receptor tyrosine kinase (gp145trkB) have been involved in immune-mediated neuroprotection in MS lesions [115,116]. In other situations, vessels act as guidance templates for axons, releasing guidance cues such as VEGF, artemin, neurotrophin-3 or ET-3 [117].

VEGF-Rs cooperate with the Notch pathway during vascular patterning and also neurogenesis [118]. Notch-1 and Notch-4 receptors, as well as Jagged-1, delta like 1 (Dll-1), and Dll-4 ligands of Notch, are expressed in endothelial cells [119]. In adult brain, the Notch pathway is expressed in SVZ and SGZ NSCs and regulates the maintenance of an undifferentiated state [120]. In addition, Notch–expressing NSCs are closely juxtaposed to local blood vessels, and able to directly bind Dll-4 and Jagged-1 exposed on the endothelial cells, where a decreased pericyte coverage exists [121]. MS demyelinated lesions contain Notch-expressing OPCs and modulation of the Notch pathway in EAE enhances remyelination and clinical recovery [122].

Wnt/β-catenin and Sonic HedgeHog (SHH) morphogen signallings both regulate embryonic neurogenesis and angiogenesis [123,124] and are variably associated with the remyelination process [125] and BBB integrity [126]. Nogo-A is an axonal growth inhibitor, and negative regulator of CNS angiogenesis [127]; anti-Nogo IgGs have been shown to suppress EAE through an immunomodulatory activity and the removal of remyelination obstacles between axons and new myelinating membranes [128].

Netrin-1 is a matrix-bound molecule interacting with different receptors (UNC and DCC, certain integrins, DSCAM - Down’s syndrome cell adhesion molecule and adenosine receptor AR2b) involved in axon guidance and angiogenic blood vessel guidance [reviewed in 91], that has been shown to inhibit migration of oligodendrocyte precursor cells into the demyelinated lesions [129]. Ephrins and their Eph receptors are short range axon guidance molecules, expressed in developing vessels and critical for their maintenance [reviewed in 91], that have shown different expression profiles in several CNS cytotypes of MS patients [130]. The specific receptor EphA4 has been implicated in the onset and a more severe course of EAE, probably due to increased axon damage during demyelination [131]. Also semaphorins and their receptors, plexins and neuropilins, regulate both axon guidance and angiogenic vessel branching and extension [reviewed in 91], and are crucially involved in remyelination failure in MS [132,133], dysregulation of T cell responses and the maintenance of tolerance in EAE [134,135].

Ang-1 and −2 also play an angiogenic role, together with VEGF, during blood vessel formation and stimulate proliferation and migration of neural precursor cells (NPCs) [90,136]. The expression level of Ang-2 is increased in RR-MS patients sera (Table 1) and in EAE mice spinal cord [45,64].

Erythropoietin (EPO) promotes angiogenesis, VEGF secretion and VEGF-R2 expression on the cerebral endothelium and also CNS neurogenesis, directly via the EPO receptor and indirectly via BDNF-increased secretion and/or suppression of cytokine signalling [137]. The relevant neuroprotective, proangiogenic and anti-inflammatory potential of EPO in MS/EAE is discussed below. In addition, oestrogen and androgen promote angiogenesis and neurogenesis after CNS injury [138,139]. EAE studies with various sexual hormones or estrogen receptor (ER) ligand treatments led to clinical disease protection, as well as protection against CNS inflammation, demyelination and axonal loss [reviewed in 138]. ERβ ligand may not only prevent demyelination, but also promote remyelination [140]. In a pathological situation (stroke), nitric oxide (NO) has a dual role in promoting angiogenesis and neurogenesis [141,142] and its action is closely linked to VEGF and BDNF expression in endothelial cells [143].

Recent studies have revealed that a hypoxic gradient is an adequate stimulus to foster angiogenesis and neurogenesis, upregulating HIF-1 [144]. Somatic stem cells reside within hypoxic niches, where low oxygen prevents oxidative stress and premature differentiation [145]. Moreover, NSCs have been observed to migrate to brain regions where active angiogenesis is occurring in neurological diseases [90], creating a temporary vascular niche where the angiogenesis and neuroreparative processes are reciprocally fostered [146].

**Figure 2:**
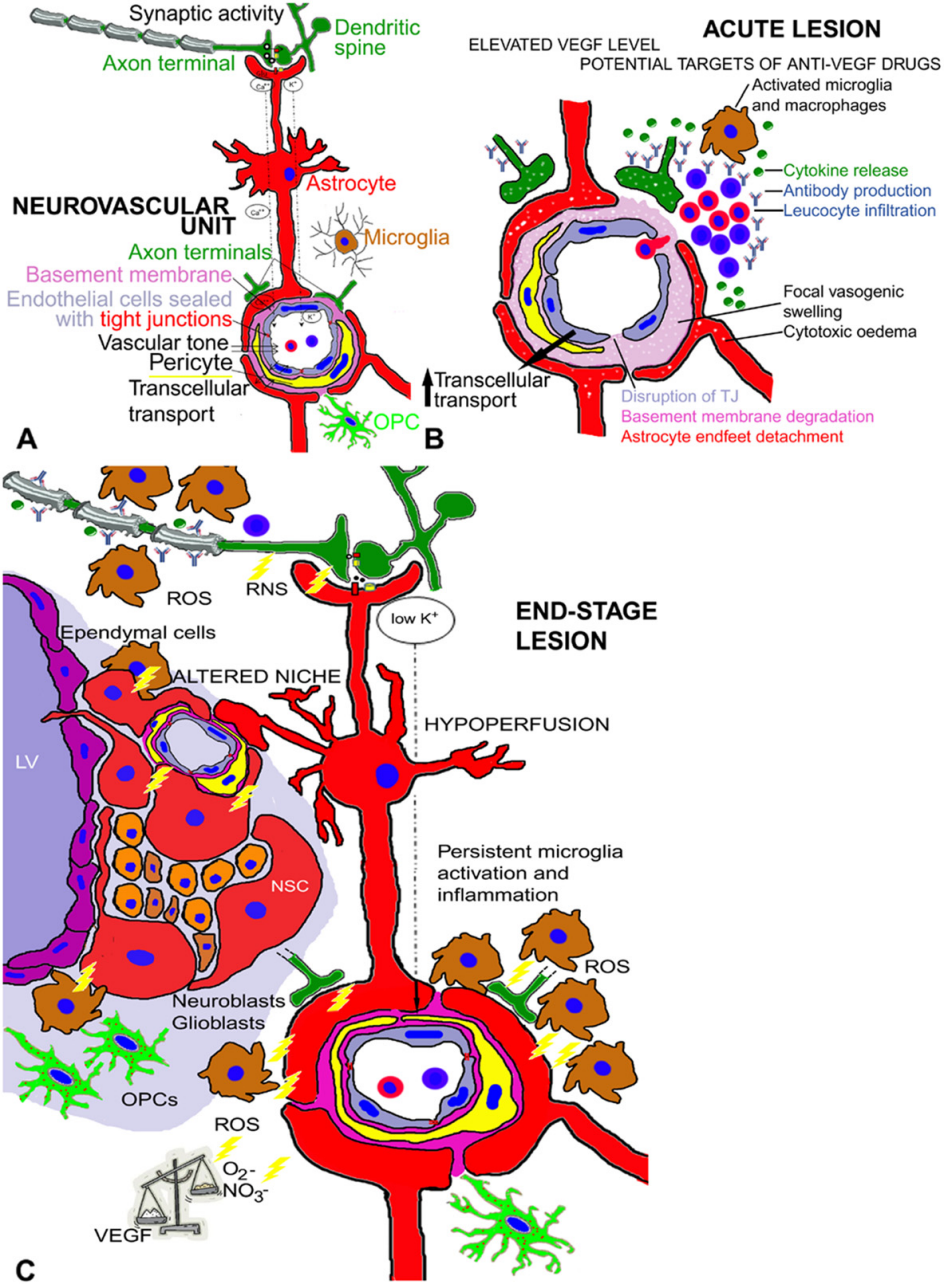
**Hypothetical model of Neurovascular Unit function (NVU) (A) and dysfunction in an acute MS lesion (B) and a chronic end-stage lesion (C).** **(A)** Composition of blood–brain barrier (BBB)-provided microvessels, formed by endothelial cells which are connected by tight junctions (TJ), pericytes, astrocyte endfeet, and a continuous layer of basement membrane (BM). Neurovascular coupling is brought about by astrocyte processes which remove excess K^+^ ions at active synaptic spaces and release these ions into perivascular spaces; at the same time Glutamate (Glu) bound to astrocyte receptors can increase astrocytic Ca^++^ levels and produce vasodilatatory substances. Microglia and oligodendrocyte precursor cells (OPCs) contribute to NVU function. **(B)** An acute MS lesion, dominated by high levels of VEGF and other angiogenic molecules, shows BBB leakiness, vasogenic swelling of BM and disrupted NVU interactions: claudin-5 and occludin, two TJ proteins, are mislocalized and downregulated; the BM is degraded by MMPs, mainly released by leukocytes infiltrating vessel BM; microglia are activated and release large amounts of pro-inflammatory mediators; astrocyte endfeet are detached from pericytes. Activated B lymphocytes release self-targeted antibodies damaging myelinated axons. **(C)** Chronic end-stage MS lesion dominated by hypoperfusion, and persistence of an inflammatory milieu with abundant reactive oxygen species (ROS), peroxynitrite (RNS) and stress-associated proteins, all together inhibiting the net pro-angiogenic activity. The drawing shows pro-inflammatory microglia and also the influence of demyelination on reduced axonal activities, decreased vasodilatatory stimuli and consequent vasoconstriction. Hypoperfusion is also due to vessel wall hyalinization, collagen deposition and astrocyte endfeet hypertrophy. Persistent inflammation is also responsible for endothelial-derived protective molecules and growth factors downregulation which, in turn, maintains neural stem cells (NSC) in a resting state and impedes neuroblast and OPC maturation in the neurovascular niches in the subependymal layer of the lateral ventricle (LV) and in other neuroregenerative sites around blood microvessels.

In the context of mutual relationships between different cells of the neurovascular unit, endothelial cells of microvessels have shown to provide trophic support for OPCs [147]. During development, OPCs migrate from the ventricular zone to their destination and then differentiate into myelinating oligodendrocytes. OPCs are also widely distributed in adult human brain and MS lesions [148] and are guided to repair demyelinated axons [149]. Endothelial cells actively support the maintenance of OPCs, acting directly through endothelin B receptors expressed by OPCs [150]. Several growth factors, such as PDGF-α, bFGF, hepatocyte growth factor (HGF), are known to induce proliferation and differentiation of OPCs, but VEGF produced by cerebral endothelial cells has a unique migration-promoting effect on OPCs [99]. Thus, VEGF is a biphasic mediator in the neurovascular response to demyelinating injury; during the early inflammatory phase it promotes BBB permeability, and in the chronic phase, accelerates not only angiogenesis, neurogenesis but also oligodendrocyte lineage plasticity and repair. In fact, exposure of endothelial cells to sublethal levels of oxidative stress abrogates their support of OPC viability [147] and this could explain why OPC differentiation into myelinating oligodendrocytes seems to be blocked or ineffective in MS. Additionally, in response to injury, activated astrocytes release bursts of ATP and induce hypertrophy of their vascular endfeet [137]. This locally increased ATP and decreased oxygen potentiates NSC expansion by upregulating VEGF, EGF, FGF-2 and NO [151] but delaying differentiation. Angiocentric perivascular demyelinated lesions show local inflammation also in the proximity of the lateral ventricles SVZ, and the effects of released inflammatory mediators on the neurovascular niches may be profound in this area, that is one of the preferential locations of demyelinating inflammatory lesions in MS [152]. Persistent brain inflammation, induced by immune cells targeting myelin, extensively alters the proliferative and migratory properties of SVZ-resident stem cells (NPCs and OPCs) [153,154], and could justify the limited repair mechanisms observed after a long disease duration in MS patients (Figure 2). In addition, MS CSF contains a panoply of humoral signals that could interfere with the ependymal cells and consequently the subependymal neurogenic cells [155].

## Chronic hypoperfusion, hypoxia and angiogenesis

Positron Emission Tomography (PET) and Single Photon Emission Computed Tomography (SPECT) studies have shown a decreased cerebral blood flow (CBF) in grey and white matter of MS patients [156,157]. Non conventional magnetic resonance (MR) techniques, such as proton MR spectroscopy and magnetization transfer resonance, have demonstrated diffuse pathological changes affecting both NAWM and NAGM in MS patients*.* Perfusion weighted imaging showed a significant CBF reduction and prolonged transit time throughout the NAWM of a group of RR-MS patients [158], and also involving NAGM [159]. Furthermore, CBF and cerebral blood volume (CBV) were reduced in primary progressive (PP-MS) patients [160,161]. A decreased blood flow has been speculatively proposed as a cause of leukocyte infiltration crossing the venule wall of WM [162] but CNS hypoperfusion could actually be a consequence of disease progression.

Acute lesions visible as local gd.e. areas on T1 weighted MRI were characterized by increased CBF and CBV [163,164]. However, more evolved MR parameters for nervous tissue angiogenesis such as time-dependent changes in 1/T_1_ (R_1_), used to form maps of blood-to-brain transfer constant of Gd-DTPA (K_i_), ICAM-1 micron-sized particles of iron oxides, in addition to magnetization transfer parameters such as T_1sat_ and k_inv_ [165], could be used to further investigate MS angiogenesis *in vivo*. Our opinion is that BBB incompetence, demonstrated by gd.e., could reveal the same MRI sign both in early, immature angiogenic microvessels and in inflamed venules. In fact, possible evidence of the presence of angiogenesis in MS could be the appearance of “ring enhancement” at the periphery, but not in the centre, of chronic active lesions during contrast-enhanced MRI [166]. Nevertheless, ring enhancing lesions are unusual in progressive MS and, in general, gd.e. is able to detect venular BBB incompetence in acute MS lesions containing both early angiogenic vessels and infiltration of immune cells.

Chronic lesions and the remaining NAWM and NAGM appeared hypoperfused due to a reduced axonal activity [167], with a lower K^+^ release in the periaxonal and perivascular space, reduced astrocyte metabolism [168] and reduced arteriolar vasodilatation [169] (Figure 2C). In this context it is not surprising to find elevated VEGF signalling [58], increased vessel density and angiogenic endothelial cells in MS chronic demyelinated lesions and NAWM [22] as a frustrated attempt to overcome the chronic hypoperfusion.

In short, angiogenesis and an increased vascular blood flow could dominate the early inflammatory phase of lesion formation, whereas, despite an increased vessel density, hypoperfusion could characterize the late degenerative phase, featuring a limited efficiency of endogenous neuroprotective mechanisms, by which angiogenesis, increased cerebral blood flow and neurorepair should be further promoted. This notion could be extended, since raised perfusion was higher in the WM of RR-MS at onset, before therapy, whereas hypoperfusion was more prominent in the PP-MS group [161], consistently associated with axonal loss, minor inflammatory signs and resistance to the available immunomodulatory drugs [170].

The role of hypoxia in inflammatory lesions of both MS and EAE may be compound, since chronic mild hypoxia (10% O_2_) has a beneficial effect in the acute and chronic phases in MOG-induced mouse EAE [171]. This effect is due to the promotion of tissue survival but also to the modulation of immune mechanisms: pericytes produce anti-inflammatory eicosanoid prostaglandin D2, endothelial cells release TGF-β that promotes the differentiation of T regulatory cells, and astrocytes express HIF-1α [172]. One of the most consistent differences in gene expression between secondary progressive (SP-MS) patients and healthy controls was enhancement of HIF-1α and its downstream components [58]. In this specific inflammatory condition, the increased effort of HIF-1α and VEGF promoting angiogenesis/arteriogenesis and normalizing oxygen levels to maintain oligodendrocyte and neuron functions could be counterbalanced by other molecules, such as reactive oxygen species (ROS) [173–175], nitric oxide intermediates and peroxynitrite (RNS) [176]. These molecules could be responsible for mitochondrial dysfunction [173], distal oligodendropathy [177], apoptotic-like cell death and axonal injury [178]. In MS patients, these pathologic mechanisms are associated with astrocyte dysfunction [167–169], which could explain the arteriolar vasoconstriction in the presence of high metabolic demands (neurovascular uncoupling), accounting for so severe a hypoperfusion state as to result in hypoxia, and ultimately responsible for disease progression. In fact, the level of VEGF expression in resident astrocytes and neurons appears increased in progressive MS patients [35,58], as well as in RR-MS [35], and a reduced level of VEGF has been detected only in non-resident mononuclear cells in CSF and peripheral blood [53,63]. This latter evidence could be compatible with the hypothesis of a true histotoxic hypoxia, as well as the observed lactate increase in CSF and serum of MS patients [179,180]. Another important pathogenic aspect of chronic progressive MS could be mitochondrial dysfunction, aggravating the nervous tissue distress caused by hypoxic injury [177]. Inflammatory cells (especially macrophages and activated microglia) releasing ROS and RNS [173,176,181] provoke clonally expanded mitochondrial DNA deletions responsible for respiratory chain defects detected in MS patients [182,183] and a consequently inevitably decreased ATP synthesis.

Finally, cerebral hypoperfusion in MS patients might be aggravated by ET-1 [85,184,185], together with alterations in the renin-angiotensin-aldosterone-system detected in MS patients such as decreased CSF angiotensin II levels [186], increased serum angiotensinogen converting enzyme [187] and up-regulation of angiotensin II receptor type I on myelin-autoreactive CD4^+^ T cells and monocytes of MS brain lesions [188].

## Therapeutic potential of targeting angiogenesis

Although angiogenesis is likely not the first event in the pathogenesis of MS, its changing role in the different phases of disease progression makes it an important and underestimated target in therapeutic options. The current concept of the natural history of MS refers to a combination of two phenomena underlying the two phases of MS, namely an inflammatory process in the remitting phase and a neurodegenerative process in the progressive phase. The secondary progressive phase of MS is primarily caused by axonal degeneration following demyelination. The potential advantages of inhibiting angiogenesis in the early phase of MS could stem from reducing the vascular supply of nutrients and inflammatory cells to the demyelinating lesions, halting the production of endothelial-derived pro-inflammatory molecules [189]. This approach could be proposed only in aggressive acute relapsing MS, where immunosuppression could be associated with specific antiangiogenic therapy. Considering the central role of VEGF signalling in pathological angiogenesis during the early MS phase, anti-VEGF therapy should be highly beneficial in the aggressive MS-subtype. We will briefly discuss some of these strategies, but do not propose to provide an exhaustive review of the literature.

Bevacizumab, a monoclonal anti-VEGF antibody approved for renal, ovarian, lung and mammary glands malignancies, that has been proven to ameliorate EAE [64], is now being tested in a clinical trial in a group of patients with neuromyelitis optica, an aggressive disease mimicking MS (ClinicalTrials.gov Identifier: NCT01777412). Nevertheless, experimental inhibition of VEGF signalling using another neutralizing antibody decreased angiogenesis and astroglial proliferation, but led to greater neurodegeneration in a model of stab wound injury of the CNS [190]. In murine MOG-EAE, antagonizing VEGF-R2 with Semaxinib (SU5416) was effective only in the acute inflammatory phase of the disease, but not in the chronic, degenerative phase [46]. In addition, Bouerat et al. [191] demonstrated a high efficacy of several anti-VEGF-R2 analogues and pro-drugs in an EAE model. Systemic administration of cavtratin, a selective eNOS inhibitor that can abrogate VEGF signalling, reverted neurologic deficits in EAE mice [39].

Bortezomib, a proteasome inhibitor, could be useful to treat MS considering its potent anti-lymphocytic and anti-angiogenic activity [192].

Thalidomide, and its analogue lenalidomide, are known to inhibit TNF-α, VEGF and IL-6 production [193]. Thus, the use of thalidomide in MS has been suggested, considering its protective action against endothelial damage induced by TNF-α [194,195], reduced leukocyte chemotaxis and phagocyte activity [196], inhibition of IFN-γ and IL-12 [197], co-stimulation of CD8+ lymphocytes [198]. Thalidomide has been demonstrated to restore BBB tightness and to protect the CNS in two experimental models of brain toxicity [199]. In the MOG-induced EAE model, N-(aminopropyl)-4-amino thalidomide is a promising therapeutic tool, able to reverse clinical and histological signs of EAE [36,200].

Corticosteroids stabilize the BBB [201] and inhibit angiogenesis in tumours [202] and chronic inflammation [203] (Table 2).

**Table 2:** **Currently used disease-modifying agents and acute exacerbation medications with an anti-angiogenic property**

**Chemical name**	**Brand name**	**References related to anti-angiogenic activity**
Alemtuzumab^1^	Lemtrada	[213]
Cyclophosphamide^2^	Endoxan, Cytoxan, Neosar, Procytox, Revimmune	[217]
Dexamethasone	Decadron	[203]
Methylprednisolone	Solu-Medrol	[203]
Dimethyl fumarate	Tecfidera	[215]
Fingolimod	Gilenya	[210–212]
Glatiramer acetate	Copaxone	[206,207]
Interferon β-1a	Avonex, Rebif	[84,204,205]
Interferon β-1b	Betaferon, Extavia	
Mitoxantrone	Novantrone	[216]
Natalizumab	Tysabri	[208,209]
Teriflunomide	Aubagio	Only indirect evidence derived from anti-lymphocytes activity

^1^licensed for MS therapy by the European Medicine Agency (EMA) but rejected by the Food and Drugs Administration (FDA), USA; ^2^not licensed but used in clinical practice.

IFN-β displays anti-angiogenic and BBB stabilizing properties [84,204,205]. Glatiramer Acetate (GA; copolymer 1), a mixture of synthetic peptides mimicking myelin basic protein, used as a first-line treatment option for RR-MS, inhibits a tryptophanyl-tRNA synthetase known to modulate angiogenic signalling [206,207]. The selective adhesion molecule inhibitor Natalizumab, which binds integrin-α4 on endothelial cells and blocks the VCAM-1 driven transmigration of immune cells sensitized against myelin antigen from the vessel lumen to the neuropil across the BBB, precludes VEGF-induced angiogenesis [208,209]. Fingolimod (FTY720), an immunomodulator that acts on sphingosine 1-phosphate (S1P) receptors, is the first oral drug approved for the treatment of RR-MS. Downmodulation of S1P receptor type 1 (S1P1) prevents the release of lymphocytes from lymph nodes into the lymphatic vessels and vascular recirculation to the CNS, reduces astrogliosis, restores BBB function, and inhibits angiogenesis during chronic neuroinflammation, also via inhibiting PDGF-B-induced migration of vascular smooth muscle cells [210–212]. Alemtuzumab, recently licensed for the treatment of MS, is a humanized monoclonal antibody directed against CD52, a protein that is widely distributed on the surface of lymphocytes and monocytes and is also an anti-angiogenic molecule [213]. Teriflunomide, an inhibitor of the mitochondrial enzyme dihydroorotate dehydrogenase, which is critically involved in pyrimidine synthesis, inhibits immune cell proliferation but shows only an indirect antiangiogenic activity. Dimethyl fumarate is the active compound of BG-12, recently licensed for the treatment of RR-MS; its activity is predominantly mediated via activation of the nuclear 1 factor (erythroid-derived 2)–like 2 (Nrf2) antioxidant response pathway [214]. BG-12 also modulates immune-cell responses, suppresses proinflammatory-cytokine production and inhibits angiogenesis [215] (Table 2).

Immunosuppressive therapies (i.e. mitoxantrone, cyclophosphamide), used to revert the aggressive course of MS, also exert an anti-angiogenic activity [216,217]. A chemotherapeutic agent, cladribine, effective but unsafe in MS, decreases the level of angiogenic factors [218]. Mycophenolate mofetil is an immunosuppressive agent, sometimes used as a disease-modifying therapy for MS, that can stabilize aggressive MS patients, and shows an anti-angiogenic activity [219]. Minocycline has been effective in EAE [220]; it is an anti-angiogenic drug in tumours [221], decreases VEGF and MMP-9 [222,223] and has been tested in combination with IFN-β (NCT01134627) and GA (NCT00203112) [224].

To date, disease modifying drugs have been shown to have little impact on the natural course of the progressive phase of MS. The development of add-on treatments targeting axonal repair and remyelination and/or slowing disease progression through neuroprotection/neuroregeneration remains the most important goal in the clinical management of chronic progressive MS [225,226]. As the endogenous neuroregenerative response can be suppressed by inflammation or exhaustion, delivery of neurovascular factors by mesenchymal, foetal or bone marrow-derived stem cells could increase endogenous repair, angiogenesis, neuronal and axonal survival and oligodendrocyte maturation and myelin synthesis. The multitasking vascular and neuroprotective effects of VEGF show promise for therapeutic use in neurodegenerative disorders such as ALS, PD, AD and, eventually, progressive MS [227–229], when its harmful vascular side effects can be restricted. Intracerebroventricular delivery of recombinant VEGF protein improves motor performance and survival in a rodent model of ALS [230]. In a stroke model, exogenous VEGF administration increases neurogenesis of the SVZ, only after 28 days, without concomitant angiogenesis, demonstrating that a specific VEGF isoform could protect neurons independently of the endothelial cell influence [231]. In the EAE model, despite several reports of an improved clinical score after early VEGF inhibition, one study [232] demonstrated that pertussis toxin stimulated VEGF expression and that VEGF neuroprotection could be responsible for milder disease. VEGF may have different effects in different cell types depending on different splice variants [233]. The endogenous splice isoform VEGF-A_165_b has shown a potent neuroprotective effect in hippocampal and cerebro-cortical neurons (mediated by VEGFR2 and neuropilin-1 co-stimulation) with no pro-permeability property [78,234]. This isoform may be an interesting add-on therapy option against axon damage in progressive MS, with fewer adverse vascular effects. Another interesting approach could be to specifically inhibit vascular permeability without affecting the endogenous neuroprotective effect of VEGF. This approach has been successfully investigated in EAE mice using angiostatin [235], cavtratin [39], anti-microRNA-155 [236] and needs to be replicated in humans.

A protective effect of vitamin D on the risk of MS has been demonstrated [237] and several trials suggested beneficial effects of vitamin D supplementation. Vitamin D3 promotes angiogenesis in endothelial cell cultures [238]. Atorvastatin, pravastatin and simvastatin have both anti- and pro-angiogenic activities depending on the dose, specific angiogenic stimulus, and angiogenesis mechanism in the specific disease local microenviroment [239,240]. They have been tested as neurorepair attempts in several randomized clinical trials in combination with IFN-β and GA [241–244].

Because neurons, oligodendrocytes and blood vessels are involved in the pathogenesis of MS, it would be better to use the same compound to treat all involved systems. Apart from VEGF, other molecules can stimulate neurogenesis, oligodendrogenesis and angiogenesis. The first is thyroxine (T4), that can decrease EAE severity [245] increase NGF and promote neurogenesis and remyelination. Other potential treatment options in selected MS patients could be sexual hormones added to an immunomodulator [138]. Among potential candidate compounds for neuroprotection/neuroregeneration and angiogenesis modulators in progressive MS, EPO appears very promising. EPO possesses properties that could address several of the pathophysiological mechanisms involved in progressive MS, being an anti-apoptotic and anti-oxidative molecule, promoting neurite outgrowth and axonal repair, neurogenesis, angiogenesis and BBB integrity (reviewed in [246]). In addition, EPO treatment could temporarily decrease iron stores within the CNS, possibly providing an additional beneficial effect in chronic progressive MS patients. Excess iron may have several deleterious effects on axons, including iron-catalyzed production of ROS and RNS causing oxidative tissue injury. Iron accumulation may also alter oligodendrocyte activities (reviewed in [246]). Recombinant human EPO treatment has already proven safe and effective in severely affected MS patients [247–249]. Testing in clinical trials of EPO variants developed to minimize the risk of thromboembolism is a promising research field.

## Concluding remarks

In MS as well as in EAE, CNS lesions and surrounding NAWM/NAGM are characterized by different vascular changes in the different disease phases. In the acute demyelinating phase, there is a complex balance between vessel modulators released by inflammatory cells and hypoxia of more distant nervous tissue from blood microvessels that could be affected by localized vasogenic swelling due to the VEGF-induced altered vascular permeability [250,251]. A reduced axon activity could cause hypoperfusion and hypoxia also in the chronic disease phase (Figure 2).

Blocking VEGF signalling and angiogenesis reduced clinical and pathological signs of disease in the early phase in an animal model of MS [39,46,64,190,191]. EAE model experiments have shown that hypoxic pre-conditioning reduced the clinical severity and leukocyte infiltration thanks to increased levels of VEGF, TGF-β, IL-10 [171,172]. However, aberrant angiogenesis and localized regression of the microvasculature can contribute to brain hypoperfusion and neurovascular uncoupling [252]. In this context, the timing of vascular remodelling and growth factors release could be crucial. In early demyelinating lesions, remodelling is harmful and exacerbates the disease. Nevertheless, in chronic disease phases, angiogenesis, and especially the neuroprotective properties of VEGF, might be highly beneficial. An alternative therapeutic agent for this neurodegenerative condition with a lesser influence on cell types other than neurons, and also lacking pro-permeability/angiogenic properties, may be VEGF-A_165_b [78,234].

Angiogenesis, induced either by CNS inflammation or by hypoxia, provides trophic factors for tissue remodelling [91,253]. In a chronic hypoxia model of cerebrovascular disease, angiogenesis proceeds in the absence of BBB leakage, being associated with increased tight junction protein expression [254]; this demonstrates that angiogenesis is not indissolubly linked to BBB breakdown. In addition, resolution of impeded angiogenesis in neural stem cell niches in the SVZ would increase oxygen levels and could also promote differentiation of oligodendrocyte precursors.

Future therapeutic efforts should be based less on a total block of angiogenesis, and more on titration of the response to produce new vessels with a functional integrity. These therapeutic options could be promising for application in MS, even if the angiogenic component of MS has still to be fully explained. To determine whether there is a correlation between clinical benefit and levels of angiogenic molecules, studies comparing clinical signs and circulating angiogenic markers in treated or untreated MS patients over time are currently ongoing, together with studies exploring angiogenesis-promoting molecules versus antiangiogenic drugs in late stage chronic MOG-induced EAE. In addition, clinical trials exploring combination therapy with an MS subtype-oriented immunomodulator/immunosuppressive agent added to an angiogenic/neuroreparative molecule during the progressive phase of MS could be warranted.

## Abbreviations

AD: Alzheimer’s disease
ADAM17: Disintegrin and metalloproteinase domain-containing protein 17
ALS: Amyotrophic lateral sclerosis
Ang: Angiopoietin
AR: Adenosine receptor
ATP: Adenosine triphosphate
BBB: Blood–Brain Barrier
BDNF: Brain-derived neurotrophic factor
bFGF: Basic Fibroblast Growth Factor
BM: Basement membrane
Ca: Calcium
CBF: Cerebral blood flow
CBV: Cerebral blood volume
CD: Cluster of differentiation
CNS: Central Nervous System
cs: Clinical score
CSF: Cerebrospinal fluid
ctrl: Control
Cx43: Connexin43
CXCR4: Chemokine (C-X-C motif) receptor 4
Dll: Delta like
dpi: Days post-immunization
DSCAM: Down’s syndrome cell adhesion molecule
EAE: Experimental allergic encephalomyelitis
ECM: Extacellular matrix molecule
EGF: Epidermal Growth Factor
ENOS: endothelial Nitric Oxide Synthetase
EPO: Erythropoietin
ER: Estrogen receptor
ET: Endothelin
GA: Glatiramer acetate
gd.e.: Gadolinium-DTPA enhancement
Glu: Glutamate
HGF: Hepatocyte Growth Factor
HIF: Hypoxia Inducible Factor
ICAM-1: Intercellular adhesion molecule 1
IFN: Interferon
IgG: Immunoglobulin G
IL: Interleukin
LV: Lateral ventricle
MBP: Myelin basic protein
MHC: Major Histocompatibility Complex
MIP-1α: Macrophage inflammatory proteins 1α
MOG: Myelin Oligodendrocyte Glycoprotein
MRI: Magnetic resonance imaging
MS: Multiple sclerosis
NAGM: Normal-appearing grey matter
NAWM: Normal-appearing white matter
NGF: Nerve Growth Factor
NO: Nitric oxide
NPC: Neural precursor cell
Nrf2: Nuclear 1 factor (erythroid-derived 2)–like 2
NSC: Neural stem cells
NVU: Neurovascular unit
OPC: Oligodendrocyte precursor cell
PBMC: Peripheral blood mononuclear cells
PD: Parkinson’s disease
PDGF: Platelet-Derived Growth Factor
PECAM-1: Platelet endothelial cell adhesion molecule-1
PEDF: Pigment Epithelium-Derived Factor
PET: Positron emission tomography
PP-MS: Primary progressive MS patients
RNA: Ribonucleic acid
RNS: Reactive nitrogen species
ROS: Reactive oxygen species
RR-MS: Relapsing remitting MS
S1P: Sphingosine 1-phosphate
SDF1: Stromal cell-Derived Factor 1
SGZ: Subgranular zone
SHH: Sonic HedgeHog
SPECT: Single photon emission computed tomography
SP-MS: Secondary progressive MS patients
SVZ: Subventricular zone
T4: Thyroxine
TGF: Transforming Growth Factor
TJ: Tight junction
TNF: Tumour necrosis factor
tRNA: transfer ribonucleic acid
VCAM: Vascular cell adhesion protein 1
VEGF: Vascular Endothelial Growth Factor
VEGF-R: Vascular Endothelial Growth Factor Receptor
WM: White matter

## References

[1] Brück W, Bitsch A, Kolenda H, Brück Y, Stiefel M, Lassmann H (1997). Inflammatory central nervous system demyelination: correlation of magnetic resonance imaging findings with lesion pathology. Ann Neurol.

[2] Kermode AG, Thompson AJ, Tofts P, MacManus DG, Kendall BE, Kingsley DP, Moseley IF, Rudge P, McDonald WI (1990). Breakdown of the blood–brain barrier precedes symptoms and other MRI signs of new lesions in multiple sclerosis. Pathogenetic and clinical implications. Brain.

[3] Alvarez JI, Cayrol R, Prat A (2011). Disruption of central nervous system barriers in multiple sclerosis. Biochim Biophys Acta.

[4] Claudio L, Raine CS, Brosnan CF (1995). Evidence of persistent blood–brain barrier abnormalities in chronic-progressive multiple sclerosis. Acta Neuropathol.

[5] Kirk J, Plumb J, Mirakhur M, McQuaid S (2003). Tight junctional abnormality in multiple sclerosis white matter affects all calibers of vessel and is associated with blood–brain barrier leakage and active demyelination. J Pathol.

[6] McQuaid S, Kirk JT (2005). The blood–brain barrier in multiple sclerosis. Int Congress Series.

[7] Minagar A, Alexander JS (2003). Blood–brain barrier disruption in multiple sclerosis. Mult Scler.

[8] Plumb J, McQuaid S, Mirakhur M, Kirk J (2002). Abnormal endothelial tight junctions in active lesions and normal-appearing white matter in multiple sclerosis. Brain Pathol.

[9] van Horssen J, Bö L, Vos CM, Virtanen I, de Vries HE (2005). Basement membrane proteins in multiple sclerosis-associated inflammatory cuffs: Potential role in influx and transport of leukocytes. J Neuropathol Exp Neurol.

[10] Wosik K, Cayrol R, Dodelet-Devillers A, Berthelet F, Bernard M, Moumdjian R, Bouthillier A, Reudelhuber TL, Prat A (2007). Angiotensin II controls occludin function and is required for blood brain barrier maintenance: relevance to multiple sclerosis. J Neurosci.

[11] Gay D, Esiri M (1991). Blood–brain barrier damage in acute multiple sclerosis plaques. An immunocytological study. Brain.

[12] Su JJ, Osoegawa M, Matsuoka T, Minohara M, Tanaka M, Ishizu T, Mihara F, Taniwaki T, Kira J (2006). Upregulation of vascular growth factors in multiple sclerosis: correlation with MRI findings. J Neurol Sci.

[13] Dousset V, Brochet B, Deloire MS, Lagoarde L, Barroso B, Caille JM, Petry KG (2006). MR imaging of relapsing multiple sclerosis patients using ultra-small-particle iron oxide and compared with gadolinium. Am J Neuroradiol.

[14] Vellinga MM, Engberink RD, Seewann A, Pouwels P, Wattjes M, van der Pol S, Pering C, Polman CH, de Vries HE, Geurts JJ, Barkhof F (2008). Pluriformity of inflammation in multiple sclerosis shown by ultra-small iron oxide particle enhancement. Brain.

[15] Ladewig G, Jestaedt L, Misselwitz B, Solymosi L, Toyka K, Bendszus M, Stoll G (2009). Spatial diversity of blood–brain barrier alteration and macrophage invasion in experimental autoimmune encephalomyelitis: a comparative MRI study. Exp Neurol.

[16] Mayhan WG (1999). VEGF increases permeability of the blood–brain barrier via a nitric oxide synthase/cGMP-dependent pathway. Am J Physiol.

[17] Barleon B, Sozzani S, Zhou D, Weich HA, Mantovani A, Marmé D (1996). Migration of human monocytes in response to vascular endothelial growth factor (VEGF) is mediated via the VEGF receptor flt-1. Blood.

[18] Clauss M, Gerlach M, Gerlach H, Brett J, Wang F, Familletti PC, Pan YC, Olander JV, Connolly DT, Stern D (1990). Vascular permeability factor: a tumor-derived polypeptide that induces endothelial cell and monocyte procoagulant activity, and promotes monocyte migration. J Exp Med.

[19] Proescholdt MA, Heiss JD, Walbridge S, Mühlhauser J, Capogrossi MC, Oldfield EH, Merrill MJ (1999). Vascular endothelial growth factor (VEGF) modulates vascular permeability and inflammation in rat brain. J Neuropathol Exp Neurol.

[20] Virgintino D, Girolamo F, Errede M, Capobianco C, Robertson D, Stallcup WB, Perris R, Roncali L (2007). An intimate interplay between precocious, migrating pericytes and endothelial cells governs human fetal brain angiogenesis. Angiogenesis.

[21] Sung HK, Michael IP, Nagy A (2010). Multifaceted role of vascular endothelial growth factor signaling in adult tissue physiology: an emerging concept with clinical implications. Curr Opin Hematol.

[22] Holley JE, Newcombe J, Whatmore JL, Gutowski NJ (2010). Increased blood vessel density and endothelial cell proliferation in multiple sclerosis cerebral white matter. Neurosci Lett.

[23] Ludwin S (2001). Vascular proliferation and angiogenesis in MS: clinical and pathogenic implications. J Neuropath Exp Neurol.

[24] Vagnucci AH, Li WW (2003). Alzheimer's disease and angiogenesis. Lancet.

[25] Desai Bradaric B, Patel A, Schneider JA, Carvey PM, Hendey B (2012). Evidence for angiogenesis in Parkinson's disease, incidental Lewy body disease, and progressive supranuclear palsy. J Neural Transm.

[26] Plate KH, Scholz A, Dumont DJ (2012). Tumor angiogenesis and anti-angiogenic therapy in malignant gliomas revisited. Acta Neuropathol.

[27] Xiong M, Elson G, Legarda D, Leibovich SJ (1998). Production of vascular endothelial growth factor by murine macrophages - regulation by hypoxia, lactate, and the inducible nitric oxide synthase pathway. Am J Pathol.

[28] Papadaki EZ, Simos PG, Mastorodemos VC, Panou T, Maris TG, Karantanas AH, Plaitakis A (2014). Regional MRI perfusion measures predict motor/executive function in patients with Clinically Isolated Syndrome. Behav Neurol.

[29] Fog T (1965). The topography of plaques in multiple sclerosis. With special reference to cerebral plaques. Acta Neurol Scand.

[30] Macchi G (1954). The pathology of the blood vessels in multiple sclerosis. J Neuropath Exp Neurol.

[31] Putnam TJ (1933). The pathogenesis of multiple sclerosis: a possible vascular factor. N Engl J Med.

[32] Rindfleisch E (1863). Histologische Details zu der grauen degeneration von Gehirn und Rückenmark. Virchow’s Archiv für Pathologische Anatomie und Physiologie und für klinische Medizin.

[33] Scheinker M (1943). Histogenesis of the early lesions of multiple sclerosis. 1. Significance of the vascular changes. Arch Neurol Psychiat.

[34] Broman T (1947). Supravital analysis of disorders in the cerebral vascular permeability II. Two cases of multiple sclerosis. Acta Psychiatr Neurol Scand (Suppl).

[35] Proescholdt MA, Jacobson S, Tresser N, Oldfield EH, Merrill MJ (2002). Vascular endothelial growth factor is expressed in multiple sclerosis plaques and can induce inflammatory lesions in experimental allergic encephalomyelitis rats. J Neuropathol Exp Neurol.

[36] Karlik SJ, Roscoe WA, Patinote C, Contino-Pepin C (2012). Targeting vascular changes in lesions in multiple sclerosis and experimental autoimmune encephalomyelitis. Cent Nerv Syst Agents Med Chem.

[37] Kirk S, Frank JA, Karlik S (2004). Angiogenesis in multiple sclerosis: is it good, bad or an epiphenomenon?. J Neurol Sci.

[38] Trapp BD, Stys PK (2009). Virtual hypoxia and chronic necrosis of demyelinated axons in multiple sclerosis. Lancet Neurol.

[39] Argaw AT, Asp L, Zhang J, Navrazhina K, Pham T, Mariani JN, Mahase S, Dutta DJ, Seto J, Kramer EG, Ferrara N, Sofroniew MV, John GR (2012). Astrocyte-derived VEGF-A drives blood–brain barrier disruption in CNS inflammatory disease. J Clin Invest.

[40] Boroujerdi A, Welser-Alves JV, Milner R (2013). Extensive vascular remodeling in the spinal cord of pre-symptomatic experimental autoimmune encephalomyelitis mice; increased vessel expression of fibronectin and the α5β1 integrin. Exp Neurol.

[41] Daniel PM, Lam DK, Pratt OE (1981). Changes in the effectiveness of the blood–brain and blood-spinal cord barriers in experimental allergic encephalomyelitis. J Neurol Sci.

[42] Errede M, Girolamo F, Ferrara G, Strippoli M, Morando S, Boldrin V, Rizzi M, Uccelli A, Perris R, Bendotti C, Salmona M, Roncali L, Virgintino D (2012). Blood–brain barrier alterations in the cerebral cortex in experimental autoimmune encephalomyelitis. J Neuropathol Exp Neurol.

[43] Juhler M, Barry DI, Offner H, Konat G, Klinken L, Paulson OB (1984). Blood–brain and blood-spinal cord barrier permeability during the course of experimental allergic encephalomyelitis in the rat. Brain Res.

[44] Zlokovic BV, Skundric DS, Segal MB, Colover J, Jankov RM, Pejnovic N, Lackovic V, Mackic J, Lipovac MN, Dawson H, Kasp E, Dumonde D, Rakic L (1989). Blood–brain barrier permeability changes during acute allergic encephalomyelitis induced in the guinea pig. Metab Brain Dis.

[45] MacMillan CJ, Starkey RJ, Easton AS (2011). Angiogenesis is regulated by angiopoietins during experimental autoimmune encephalomyelitis and is indirectly related to vascular permeability. J Neuropathol Exp Neurol.

[46] Roscoe WA, Welsh ME, Carter DE, Karlik SJ (2009). VEGF and angiogenesis in acute and chronic MOG((35–55)) peptide induced EAE. J Neuroimmunol.

[47] Kirk SL, Karlik SJ (2003). VEGF and vascular changes in chronic neuroinflammation. J Autoimmun.

[48] Piraino PS, Yednock TA, Messersmith EK, Pleiss MA, Freedman SB, Hammond RR, Karlik SJ (2005). Spontaneous remyelination following prolonged inhibition of alpha4 integrin in chronic EAE. J Neuroimmunol.

[49] Sobel RA, Blanchette BW, Bhan AK, Colvin RB (1984). The immunopathology of experimental allergic encephalomyelitis. II. Endothelial cell Ia increases prior to inflammatory cell infiltration. J Immunol.

[50] Seabrook TJ, Littlewood-Evans A, Brinkmann V, Pöllinger B, Schnell C, Hiestand PC (2010). Angiogenesis is present in experimental autoimmune encephalomyelitis and pro-angiogenic factors are increased in multiple sclerosis lesions. J Neuroinflammation.

[51] Mor F, Quintana FJ, Cohen IR (2004). Angiogenesis-inflammation cross-talk: vascular endothelial growth factor is secreted by activated T cells and induces Th1 polarization. J Immunol.

[52] Sasaki M, Lankford KL, Brown RJ, Ruddle NH, Kocsis JD (2010). Focal experimental autoimmune encephalomyelitis in the Lewis rat induced by immunization with myelin oligodendrocyte glycoprotein and intraspinal injection of vascular endothelial growth factor. Glia.

[53] Tham E, Gielen AW, Khademi M, Martin C, Piehl F (2006). Decreased expression of VEGF-A in rat experimental autoimmune encephalomyelitis and in cerebrospinal fluid mononuclear cells from patients with multiple sclerosis. Scand J Immunol.

[54] McCloskey DP, Tana MH, Scharfman HE (2008). Modulation of vascular endothelial growth factor (VEGF) expression in motor neurons and its electrophysiological effects. Brain Res Bull.

[55] Knizetova P, Ehrmann J, Hlobilkova A, Vancova I, Kalita O, Kolar Z, Bartek J (2008). Autocrine regulation of glioblastoma cell cycle progression, viability and radioresistance through the VEGF-VEGFR2 (KDR) interplay. Cell Cycle.

[56] Storkebaum E, Lambrechts D, Carmeliet P (2004). VEGF: once regarded as a specific angiogenic factor, now implicated in neuroprotection. Bioessays.

[57] Zhang ZG, Zhang L, Tsang W, Soltanian-Zadeh H, Morris D, Zhang R, Goussev A, Powers C, Yeich T, Chopp M (2002). Correlation of VEGF and angiopoietin expression with disruption of blood–brain barrier and angiogenesis after focal cerebral ischemia. J Cereb Blood Flow Metab.

[58] Graumann U, Reynolds R, Steck AJ, Schaeren-Wiemers N (2003). Molecular changes in normal appearing white matter in multiple sclerosis are characteristic of neuroprotective mechanisms against hypoxic insult. Brain Pathol.

[59] Theoharides TC, Konstantinidou AD (2007). Corticotropin-releasing hormone and the blood–brain-barrier. Front Biosci.

[60] van Bruggen N, Thibodeaux H, Palmer JT, Lee WP, Fu L, Cairns B, Tumas D, Gerlai R, Williams SP, van Lookeren CM, Ferrara N (1999). VEGF antagonism reduces edema formation and tissue damage after ischemia/reperfusion injury in the mouse brain. J Clin Invest.

[61] Benton RL, Whittemore SR (2003). VEGF165 therapy exacerbates secondary damage following spinal cord injury. Neurochem Res.

[62] Widenfalk J, Lipson A, Jubran M, Hofstetter C, Ebendal T, Cao Y, Olson L (2003). Vascular endothelial growth factor improves functional outcome and decreases secondary degeneration in experimental spinal cord contusion injury. Neuroscience.

[63] Iacobaeus E, Amoudruz P, Ström M, Khademi M, Brundin L, Hillert J, Kockum I, Malmström V, Olsson T, Tham E, Piehl F (2011). The expression of VEGF-A is down regulated in peripheral blood mononuclear cells of patients with secondary progressive multiple sclerosis. PLoS One.

[64] MacMillan CJ, Furlong SJ, Doucette CD, Chen PL, Hoskin DW, Easton AS (2012). Bevacizumab diminishes experimental autoimmune encephalomyelitis by inhibiting spinal cord angiogenesis and reducing peripheral T-cell responses. J Neuropathol Exp Neurol.

[65] Lambrechts D, Storkebaum E, Morimoto M, Del-Favero J, Desmet F, Marklund SL, Wyns S, Thijs V, Andersson J, van Marion I, Al-Chalabi A, Bornes S, Musson R, Hansen V, Beckman L, Adolfsson R, Pall HS, Prats H, Vermeire S, Rutgeerts P, Katayama S, Awata T, Leigh N, Lang-Lazdunski L, Dewerchin M, Shaw C, Moons L, Vlietinck R, Morrison KE, Robberecht W, Van Broeckhoven C (2003). VEGF is a modifier of amyotrophic lateral sclerosis in mice and humans and protects motoneurons against ischemic death. Nat Genet.

[66] Oosthuyse B, Moons L, Storkebaum E, Beck H, Nuyens D, Brusselmans K, Van Dorpe J, Hellings P, Gorselink M, Heymans S, Theilmeier G, Dewerchin M, Laudenbach V, Vermylen P, Raat H, Acker T, Vleminckx V, Van Den Bosch L, Cashman N, Fujisawa H, Drost MR, Sciot R, Bruyninckx F, Hicklin DJ, Ince C, Gressens P, Lupu F, Plate KH, Robberecht W, Herbert JM (2001). Deletion of the hypoxia response element in the vascular endothelial growth factor promoter causes motor neuron degeneration. Nat Genet.

[67] Storkebaum E, Lambrechts D, Dewerchin M, Moreno-Murciano MP, Appelmans S, Oh H, Van Damme P, Rutten B, Man WY, De Mol M, Wyns S, Manka D, Vermeulen K, Van Den Bosch L, Mertens N, Schmitz C, Robberecht W, Conway EM, Collen D, Moons L, Carmeliet P (2005). Treatment of motoneuron degeneration by intracerebroventricular delivery of VEGF in a rat model of ALS. Nat Neurosci.

[68] Sondell M, Lundborg G, Kanje M (1999). Vascular endothelial growth factor has neurotrophic activity and stimulates axonal outgrowth, enhancing cell survival and Schwann cell proliferation in the peripheral nervous system. J Neurosci.

[69] Argaw AT, Gurfein BT, Zhang Y, Zameer A, John GR (2009). VEGF-mediated disruption of endothelial CLN-5 promotes blood–brain barrier breakdown. Proc Natl Acad Sci U S A.

[70] Rosenstein JM, Krum JM (2004). New roles for VEGF in nervous tissue–beyond blood vessels. Exp Neurol.

[71] Okada S, Nakamura M, Katoh H, Miyao T, Shimazaki T, Ishii K, Yamane J, Yoshimura A, Iwamoto Y, Toyama Y, Okano H (2006). Conditional ablation of Stat3 or Socs3 discloses a dual role for reactive astrocytes after spinal cord injury. Nat Med.

[72] Choi JS, Kim HY, Cha JH, Choi JY, Park SI, Jeong CH, Jeun SS, Lee MY (2007). Upregulation of vascular endothelial growth factor receptors Flt-1 and Flk-1 following acute spinal cord contusion in rats. J Histochem Cytochem.

[73] Herz J, Reitmeir R, Hagen SI, Reinboth BS, Guo Z, Zechariah A, ElAli A, Doeppner TR, Bacigaluppi M, Pluchino S, Kilic U, Kilic E, Hermann DM (2012). Intracerebroventricularly delivered VEGF promotes contralesional corticorubral plasticity after focal cerebral ischemia via mechanisms involving anti-inflammatory actions. Neurobiol Dis.

[74] Heil M, Clauss M, Suzuki K, Buschmann IR, Willuweit A, Fischer S, Schaper W (2000). Vascular endothelial growth factor (VEGF) stimulates monocyte migration through endothelial monolayers via increased integrin expression. Eur J Cell Biol.

[75] Ishizu T, Osoegawa M, Mei FJ, Kikuchi H, Tanaka M, Takakura Y, Minohara M, Murai H, Mihara F, Taniwaki T, Kira J (2005). Intrathecal activation of the IL-17/IL-8 axis in opticospinal multiple sclerosis. Brain.

[76] Cho ML, Jung YO, Moon YM, Min SY, Yoon CH, Lee SH, Park SH, Cho CS, Jue DM, Kim HY (2006). Interleukin-18 induces the production of vascular endothelial growth factor (VEGF) in rheumatoid arthritis synovial fibroblasts via AP-1-dependent pathways. Immunol Lett.

[77] Suarez S, Ballmer-Hofer K (2001). VEGF transiently disrupts gap junctional communication in endothelial cells. J Cell Sci.

[78] Beazley-Long N, Hua J, Jehle T, Hulse RP, Dersch R, Lehrling C, Bevan H, Qiu Y, Lagrèze WA, Wynick D, Churchill AJ, Kehoe P, Harper SJ, Bates DO, Donaldson LF (2013). VEGF-A165b is an endogenous neuroprotective splice isoform of vascular endothelial growth factor A in vivo and in vitro. Am J Pathol.

[79] Kawamura H, Li X, Harper SJ, Bates DO, Claesson-Welsh L (2008). Vascular endothelial growth factor (VEGF)-A165b is a weak in vitro agonist for VEGF receptor-2 due to lack of coreceptor binding and deficient regulation of kinase activity. Cancer Res.

[80] Liu Y, Cox SR, Morita T, Kourembanas S (1995). Hypoxia regulates vascular endothelial growth factor gene expression in endothelial cells. Identification of a 5' enhancer. Circ Res.

[81] Lassmann H (2003). Hypoxia-like tissue injury as a component of multiple sclerosis lesions. J Neurol Sci.

[82] Distler JH, Hirth A, Kurowska-Stolarska M, Gay RE, Gay S, Distler O (2003). Angiogenic and angiostatic factors in the molecular control of angiogenesis. Q J Nucl Med.

[83] Frost EE, Nielsen JA, Le TQ, Armstrong RC (2003). PDGF and FGF2 regulate oligodendrocyte progenitor responses to demyelination. J Neurobiol.

[84] Van Meir EG (1995). Cytokines and tumors of the central nervous system. Glia.

[85] Haufschild T, Shaw SG, Kesselring J, Flammer J (2001). Increased endothelin-1 plasma levels in patients with multiple sclerosis. J Neuroophthalmol.

[86] Shin T, Kang B, Tanuma N, Matsumoto Y, Wie M, Ahn M, Kang J (2001). Intrathecal administration of endothelin-1 receptor antagonist ameliorates autoimmune encephalomyelitis in Lewis rats. Neuroreport.

[87] Li L, Welser-Alves J, van der Flier A, Boroujerdi A, Hynes RO, Milner R (2012). An angiogenic role for the α5β1 integrin in promoting endothelial cell proliferation during cerebral hypoxia. Exp Neurol.

[88] Cunnea P, McMahon J, O'Connell E, Mashayekhi K, Fitzgerald U, McQuaid S (2010). Gene expression analysis of the microvascular compartment in multiple sclerosis using laser microdissected blood vessels. Acta Neuropathol.

[89] Jackson JR, Seed MP, Kircher CH, Willoughby DA, Winkler JD (1997). The codependence of angiogenesis and chronic inflammation. FASEB J.

[90] Ward NL, Lamanna JC (2004). The neurovascular unit and its growth factors: coordinated response in the vascular and nervous systems. Neurol Res.

[91] Carmeliet P, Ruiz de Almodovar C (2013). VEGF ligands and receptors: implications in neurodevelopment and neurodegeneration. Cell Mol Life Sci.

[92] Carmeliet P, Tessier-Lavigne M (2005). Common mechanisms of nerve and blood vessel wiring. Nature.

[93] Leventhal C, Rafii S, Rafii D, Shahar A, Goldman SA (1999). Endothelial trophic support of neuronal production and recruitment from the adult mammalian subependyma. Mol Cell Neurosci.

[94] Shen Q, Goderie SK, Jin L, Karanth N, Sun Y, Abramova N, Vincent P, Pumiglia K, Temple S (2004). Endothelial cells stimulate self-renewal and expand neurogenesis of neural stem cells. Science.

[95] Zacchigna S, Lambrechts D, Carmeliet P (2008). Neurovascular signalling defects in neurodegeneration. Nat Rev Neurosci.

[96] Jin K, Zhu Y, Sun Y, Mao XO, Xie L, Greenberg DA (2002). Vascular endothelial growth factor (VEGF) stimulates neurogenesis in vitro and in vivo. Proc Natl Acad Sci U S A.

[97] Zhao C, Deng W, Gage FH (2008). Mechanisms and functional implications of adult neurogenesis. Cell.

[98] Ma YY, Li KY, Wang JJ, Huang YL, Huang Y, Sun FY (2009). Vascular endothelial growth factor acutely reduces calcium influx via inhibition of the Ca2+ channels in rat hippocampal neurons. J Neurosci Res.

[99] Hayakawa K, Pham LD, Som AT, Lee BJ, Guo S, Lo EH, Arai K (2011). Vascular endothelial growth factor regulates the migration of oligodendrocyte precursor cells. J Neurosci.

[100] Gao P, Shen F, Gabriel RA, Law D, Yang E, Yang GY, Young WL, Su H (2009). Attenuation of brain response to vascular endothelial growth factor-mediated angiogenesis and neurogenesis in aged mice. Stroke.

[101] Kazanis I, Lathia JD, Vadakkan TJ, Raborn E, Wan R, Mughal MR, Eckley DM, Sasaki T, Patton B, Mattson MP, Hirschi KK, Dickinson ME, Ffrench-Constant C (2010). Quiescence and activation of stem and precursor cell populations in the subependymal zone of the mammalian brain are associated with distinct cellular and extracellular matrix signals. J Neurosci.

[102] Kokovay E, Goderie S, Wang Y, Lotz S, Lin G, Sun Y, Roysam B, Shen Q, Temple S (2010). Adult SVZ lineage cells home to and leave the vascular niche via differential responses to SDF1/CXCR4 signaling. Cell Stem Cell.

[103] Patel JR, McCandless EE, Dorsey D, Klein RS (2010). CXCR4 promotes differentiation of oligodendrocyte progenitors and remyelination. Proc Natl Acad Sci U S A.

[104] Kerever A, Schnack J, Vellinga D, Ichikawa N, Moon C, Arikawa-Hirasawa E, Efird JT, Mercier F (2007). Novel extracellular matrix structures in the neural stem cell niche capture the neurogenic factor fibroblast growth factor 2 from the extracellular milieu. Stem Cells.

[105] Clemente D, Ortega MC, Arenzana FJ, de Castro F (2011). FGF-2 and Anosmin-1 are selectively expressed in different types of multiple sclerosis lesions. J Neurosci.

[106] Sohn J, Selvaraj V, Wakayama K, Orosco L, Lee E, Crawford SE, Guo F, Lang J, Horiuchi M, Zarbalis K, Itoh T, Deng W, Pleasure D (2012). PEDF is a novel oligodendrogenic morphogen acting on the adult SVZ and corpus callosum. J Neurosci.

[107] Aguirre A, Rubio ME, Gallo V (2010). Notch and EGFR pathway interaction regulates neural stem cell number and self-renewal. Nature.

[108] Rash BG, Lim HD, Breunig JJ, Vaccarino FM (2011). FGF signaling expands embryonic cortical surface area by regulating Notch-dependent neurogenesis. J Neurosci.

[109] Cha YK, Kim YH, Ahn YH, Koh JY (2000). Epidermal growth factor induces oxidative neuronal injury in cortical culture. J Neurochem.

[110] Levy YA, Fainberg KM, Amidror T, Regev K, Auriel E, Karni A (2013). High and dysregulated secretion of epidermal growth factor from immune cells of patients with relapsing-remitting multiple sclerosis. J Neuroimmunol.

[111] Calza L, Giuliani A, Fernandez M, Pirondi S, D’Intino G, Aloe L, Giardino L (2003). Neural stem cells and cholinergic neurons: regulation by immunolesion and treatment with mitogens, retinoic acid, and nerve growth factor. Proc Natl Acad Sci U S A.

[112] Kim H, Li Q, Hempstead BL, Madri JA (2004). Paracrine and autocrine functions of brain-derived neurotrophic factor (BDNF) and nerve growth factor (NGF) in brain-derived endothelial cells. J Biol Chem.

[113] Mashayekhi F, Salehi Z, Jamalzadeh HR (2012). Quantitative analysis of cerebrospinal fluid brain derived neurotrophic factor in the patients with multiple sclerosis. Acta Med (Hradec Kralove).

[114] Laudiero LB, Aloe L, Levi-Montalcini R, Buttinelli C, Schilter D, Gillessen S, Otten U (1992). Multiple sclerosis patients express increased levels of beta-nerve growth factor in cerebrospinal fluid. Neurosci Lett.

[115] Stadelmann C, Kerschensteiner M, Misgeld T, Brück W, Hohlfeld R, Lassmann H (2002). BDNF and gp145trkB in multiple sclerosis brain lesions: neuroprotective interactions between immune and neuronal cells?. Brain.

[116] Kalinowska-Lyszczarz A, Losy J (2012). The role of neurotrophins in multiple sclerosis-pathological and clinical implications. Int J Mol Sci.

[117] Larrivée B, Freitas C, Suchting S, Brunet I, Eichmann A (2009). Guidance of vascular development: lessons from the nervous system. Circ Res.

[118] Mizutani K, Yoon K, Dang L, Tokunaga A, Gaiano N (2007). Differential Notch signalling distinguishes neural stem cells from intermediate progenitors. Nature.

[119] Kume T (2009). Novel insights into the differential functions of Notch ligands in vascular formation. J Angiogenes Res.

[120] Shimojo H, Ohtsuka T, Kageyama R (2008). Oscillations in notch signaling regulate maintenance of neural progenitors. Neuron.

[121] Tavazoie M, Van der Veken L, Silva-Vargas V, Louissaint M, Colonna L, Zaidi B, Garcia-Verdugo JM, Doetsch F (2008). A specialized vascular niche for adult neural stem cells. Cell Stem Cell.

[122] Juryńczyk M, Selmaj K (2010). Notch: a new player in MS mechanisms. J Neuroimmunol.

[123] Hashimoto M, Ishii K, Nakamura Y, Watabe K, Kohsaka S, Akazawa C (2008). Neuroprotective effect of sonic hedgehog up-regulated in Schwann cells following sciatic nerve injury. J Neurochem.

[124] Hirsch C, Campano LM, Wohrle S, Hecht A (2007). Canonical Wnt signaling transiently stimulates proliferation and enhances neurogenesis in neonatal neural progenitor cultures. Exp Cell Res.

[125] Xie C, Li Z, Zhang GX, Guan Y (2013). Wnt signaling in Remyelination in multiple sclerosis: friend or foe?. Mol Neurobiol.

[126] Alvarez JI, Dodelet-Devillers A, Kebir H, Ifergan I, Fabre PJ, Terouz S, Sabbagh M, Wosik K, Bourbonnière L, Bernard M, van Horssen J, de Vries HE, Charron F, Prat A (2011). The Hedgehog pathway promotes blood–brain barrier integrity and CNS immune quiescence. Science.

[127] Wälchli T, Pernet V, Weinmann O, Shiu JY, Guzik-Kornacka A, Decrey G, Yüksel D, Schneider H, Vogel J, Ingber DE, Vogel V, Frei K, Schwab ME (2013). Nogo-A is a negative regulator of CNS angiogenesis. Proc Natl Acad Sci U S A.

[128] Karnezis T, Mandemakers W, McQualter JL, Zheng B, Ho PP, Jordan KA, Murray BM, Barres B, Tessier-Lavigne M, Bernard CC (2004). The neurite outgrowth inhibitor Nogo A is involved in autoimmune-mediated demyelination. Nat Neurosci.

[129] Bin JM, Rajasekharan S, Kuhlmann T, Hanes I, Marcal N, Han D, Rodrigues SP, Leong SY, Newcombe J, Antel JP, Kennedy TE (2013). Full-length and fragmented netrin-1 in multiple sclerosis plaques are inhibitors of oligodendrocyte precursor cell migration. Am J Pathol.

[130] Sobel RA (2005). Ephrin A receptors and ligands in lesions and normal-appearing white matter in multiple sclerosis. Brain Pathol.

[131] Munro KM, Dixon KJ, Gresle MM, Jonas A, Kemper D, Doherty W, Fabri LJ, Owczarek CM, Pearse M, Boyd AW, Kilpatrick TJ, Butzkueven H, Turnley AM (2013). EphA4 receptor tyrosine kinase is a modulator of onset and disease severity of experimental autoimmune encephalomyelitis (EAE). PLoS One.

[132] Syed YA, Hand E, Möbius W, Zhao C, Hofer M, Nave KA, Kotter MR (2011). Inhibition of CNS remyelination by the presence of semaphorin 3A. J Neurosci.

[133] Williams A, Piaton G, Aigrot MS, Belhadi A, Théaudin M, Petermann F, Thomas JL, Zalc B, Lubetzki C (2007). Semaphorin 3A and 3 F: key players in myelin repair in multiple sclerosis?. Brain.

[134] Okuno T, Nakatsuji Y, Kumanogoh A (2011). The role of immune semaphorins in multiple sclerosis. FEBS Lett.

[135] Solomon BD, Mueller C, Chae WJ, Alabanza LM, Bynoe MS (2011). Neuropilin-1 attenuates autoreactivity in experimental autoimmune encephalomyelitis. Proc Natl Acad Sci U S A.

[136] Liu XS, Chopp M, Zhang RL, Hozeska-Solgot A, Gregg SC, Buller B, Lu M, Zhang ZG (2009). Angiopoietin 2 mediates the differentiation and migration of neural progenitor cells in the subventricular zone after stroke. J Biol Chem.

[137] Wang L, Zhang Z, Wang Y, Zhang R, Chopp M (2004). Treatment of stroke with erythropoietin enhances neurogenesis and angiogenesis and improves neurological function in rats. Stroke.

[138] Spence RD, Voskuhl RR (2012). Neuroprotective effects of estrogens and androgens in CNS inflammation and neurodegeneration. Front Neuroendocrinol.

[139] Suzuki S, Gerhold LM, Böttner M, Rau SW, Dela Cruz C, Yang E, Zhu H, Yu J, Cashion AB, Kindy MS, Merchenthaler I, Gage FH, Wise PM (2007). Estradiol enhances neurogenesis following ischemic stroke through estrogen receptors alpha and beta. J Comp Neurol.

[140] Crawford DK, Mangiardi M, Song B, Patel R, Du S, Sofroniew MV, Voskuhl RR, Tiwari-Woodruff SK (2010). Oestrogen receptor beta ligand: a novel treatment to enhance endogenous functional remyelination. Brain.

[141] Zhang R, Wang L, Zhang L, Chen J, Zhu Z, Zhang Z, Chopp M (2003). Nitric oxide enhances angiogenesis via the synthesis of vascular endothelial growth factor and cGMP after stroke in the rat. Circ Res.

[142] Zhang R, Zhang L, Zhang Z, Wang Y, Lu M, Lapointe M, Chopp M (2001). A nitric oxide donor induces neurogenesis and reduces functional deficits after stroke in rats. Ann Neurol.

[143] Li Q, Ford MC, Lavik EB, Madri JA (2006). Modeling the neurovascular niche: VEGF- and BDNF-mediated cross-talk between neural stem cells and endothelial cells: an in vitro study. J Neurosci Res.

[144] Fong GH (2008). Mechanisms of adaptive angiogenesis to tissue hypoxia. Angiogenesis.

[145] Mohyeldin A, Garzon-Muvdi T, Quinones-Hinojosa A (2010). Oxygen in stem cell biology: a critical component of the stem cell niche. Cell Stem Cell.

[146] Imitola J, Raddassi K, Park KI, Mueller FJ, Nieto M, Teng YD, Frenkel D, Li J, Sidman RL, Walsh CA, Snyder EY, Khoury SJ (2004). Directed migration of neural stem cells to sites of CNS injury by the stromal cell-derived factor 1alpha/CXC chemokine receptor 4 pathway. Proc Natl Acad Sci U S A.

[147] Arai K, Lo EH (2009). An oligovascular niche: cerebral endothelial cells promote the survival and proliferation of oligodendrocyte precursor cells. J Neurosci.

[148] Chang A, Nishiyama A, Peterson J, Prineas J, Trapp BD (2000). NG2-positive oligodendrocyte progenitor cells in adult human brain and multiple sclerosis lesions. J Neurosci.

[149] Chang A, Tourtellotte WW, Rudick R, Trapp BD (2002). Premyelinating oligodendrocytes in chronic lesions of multiple sclerosis. N Engl J Med.

[150] Gadea A, Aguirre A, Haydar TF, Gallo V (2009). Endothelin-1 regulates oligodendrocyte development. J Neurosci.

[151] Greenberg DA, Jin K (2005). From angiogenesis to neuropathology. Nature.

[152] Sanai N, Tramontin AD, Quinones-Hinojosa A, Barbaro NM, Gupta N, Kunwar S, Lawton MT, McDermott MW, Parsa AT, Manuel-Garcia Verdugo J, Berger MS, Alvarez-Buylla A (2004). Unique astrocyte ribbon in adult human brain contains neural stem cells but lacks chain migration. Nature.

[153] Pluchino S, Muzio L, Imitola J, Deleidi M, Alfaro-Cervello C, Salani G, Porcheri C, Brambilla E, Cavasinni F, Bergamaschi A, Garcia-Verdugo JM, Comi G, Khoury SJ, Martino G (2008). Persistent inflammation alters the function of the endogenous brain stem cell compartment. Brain.

[154] Rasmussen S, Imitola J, Ayuso-Sacido A, Wang Y, Starossom SC, Kivisäkk P, Zhu B, Meyer M, Bronson RT, Garcia-Verdugo JM, Khoury SJ (2011). Reversible neural stem cell niche dysfunction in a model of multiple sclerosis. Ann Neurol.

[155] Lehtinen MK, Zappaterra MW, Chen X, Yang YJ, Hill AD, Lun M, Maynard T, Gonzalez D, Kim S, Ye P, D'Ercole AJ, Wong ET, LaMantia AS, Walsh CA (2011). The cerebrospinal fluid provides a proliferative niche for neural progenitor cells. Neuron.

[156] Sun X, Tanaka M, Kondo S, Okamoto K, Hirai S (1998). Clinical significance of reduced cerebral metabolism in multiple sclerosis: a combined PET and MRI study. Ann Nucl Med.

[157] Swank RL, Roth JG, Woody DC (1983). Cerebral blood flow and red cell delivery in normal subjects and in multiple sclerosis. Neurol Res.

[158] Law M, Saindane AM, Ge Y, Babb JS, Johnson G, Mannon LJ, Herbert J, Grossman RI (2004). Microvascular abnormality in relapsing-remitting multiple sclerosis: perfusion MR imaging findings in normal-appearing white matter. Radiology.

[159] Varga AW, Johnson G, Babb JS, Herbert J, Grossman RI, Inglese M (2009). White matter hemodynamic abnormalities precede sub-cortical gray matter changes in multiple sclerosis. J Neurol Sci.

[160] Adhya S, Johnson G, Herbert J, Jaggi H, Babb JS, Grossman RI, Inglese M (2006). Pattern of hemodynamic impairment in multiple sclerosis: dynamic susceptibility contrast perfusion MR imaging at 3.0 T. Neuroimage.

[161] Rashid W, Parkes LM, Ingle GT, Chard DT, Toosy AT, Altmann DR, Symms MR, Tofts PS, Thompson AJ, Miller DH (2004). Abnormalities of cerebral perfusion in multiple sclerosis. J Neurol Neurosurg Psychiatry.

[162] Juurlink BH (1998). The multiple sclerosis lesion: initiated by a localized hypoperfusion in a central nervous system where mechanisms allowing leukocyte infiltration are readily upregulated?. Med Hypotheses.

[163] Haselhorst R, Kappos L, Bilecen D, Scheffler K, Möri D, Radü EW, Seelig J (2000). Dynamic susceptibility contrast MR imaging of plaque development in multiple sclerosis: application of an extended blood–brain barrier leakage correction. J Magn Reson Imaging.

[164] Wuerfel J, Bellmann-Strobl J, Brunecker P, Aktas O, McFarland H, Villringer A, Zipp F (2004). Changes in cerebral perfusion precede plaque formation in multiple sclerosis: a longitudinal perfusion MRI study. Brain.

[165] Jiang Q, Zhang ZG, Ding GL, Zhang L, Ewing JR, Wang L, Zhang R, Li L, Lu M, Meng H, Arbab AS, Hu J, Li QJ, Pourabdollah Nejad DS, Athiraman H, Chopp M (2005). Investigation of neural progenitor cell induced angiogenesis after embolic stroke in rat using MRI. Neuroimage.

[166] Hiehle JF, Lenkinski RE, Grossman RI, Dousset V, Ramer KN, Schnall MD, Cohen JA, Gonzalez-Scarano F (1994). Correlation of spectroscopy and magnetization transfer imaging in the evaluation of demyelinating lesions and normal appearing white matter in multiple sclerosis. Magn Reson Med.

[167] De Keyser J, Steen C, Mostert JP, Koch MW (2008). Hypoperfusion of the cerebral white matter in multiple sclerosis: possible mechanisms and pathophysiological significance. J Cereb Blood Flow Metab.

[168] Steen C, Wilczak N, Hoogduin JM, Koch M, De Keyser J (2010). Reduced creatine kinase B activity in multiple sclerosis normal appearing white matter. PLoS One.

[169] D'haeseleer M, Cambron M, Vanopdenbosch L, De Keyser J (2011). Vascular aspects of multiple sclerosis. Lancet Neurol.

[170] Brück W, Lucchinetti C, Lassmann H (2002). The pathology of primary progressive multiple sclerosis. Mult Scler.

[171] Dore-Duffy P, Wencel M, Katyshev V, Cleary K (2011). Chronic mild hypoxia ameliorates chronic inflammatory activity in myelin oligodendrocyte glycoprotein (MOG) peptide induced experimental autoimmune encephalomyelitis (EAE). Adv Exp Med Biol.

[172] Esen N, Serkin Z, Dore-Duffy P (2013). Induction of vascular remodeling: a novel therapeutic approach in EAE. J Neurol Sci.

[173] Lu F, Selak M, O'Connor J, Croul S, Lorenzana C, Butunoi C, Kalman B (2000). Oxidative damage to mitochondrial DNA and activity of mitochondrial enzymes in chronic active lesions of multiple sclerosis. J Neurol Sci.

[174] Haider L, Fischer MT, Frischer JM, Bauer J, Höftberger R, Botond G, Esterbauer H, Binder CJ, Witztum JL, Lassmann H (2011). Oxidative damage in multiple sclerosis lesions. Brain.

[175] Gironi M, Borgiani B, Mariani E, Cursano C, Mendozzi L, Cavarretta R, Saresella M, Clerici M, Comi G, Rovaris M, Furlan R (2014). Oxidative stress is differentially present in multiple sclerosis courses, early evident, and unrelated to treatment. J Immunol Res.

[176] Bolanos JP, Almeida A, Stewart V, Peuchen S, Land JM, Clark JB, Heales SJ (1997). Nitric oxide-mediated mitochondrial damage in the brain: mechanisms and implications for neurodegenerative diseases. J Neurochem.

[177] Aboul-Enein F, Rauschka H, Kornek B, Stadelmann C, Stefferl A, Brück W, Lucchinetti C, Schmidbauer M, Jellinger K, Lassmann H (2003). Preferential loss of myelin-associated glycoprotein reflects hypoxia-like white matter damage in stroke and inflammatory brain diseases. J Neuropathol Exp Neurol.

[178] Redford EJ, Kapoor R, Smith KJ (1997). Nitric oxide donors reversibly block axonal conduction: demyelinated axons are especially susceptible. Brain.

[179] Amorini AM, Nociti V, Petzold A, Gasperini C, Quartuccio E, Lazzarino G, Di Pietro V, Belli A, Signoretti S, Vagnozzi R, Lazzarino G, Tavazzi B (2014). Serum lactate as a novel potential biomarker in multiple sclerosis. Biochim Biophys Acta.

[180] Simone IL, Federico F, Trojano M, Tortorella C, Liguori M, Giannini P, Picciola E, Natile G, Livrea P (1996). High resolution proton MR spectroscopy of cerebrospinal fluid in MS patients. Comparison with biochemical changes in demyelinating plaques. J Neurol Sci.

[181] Fischer MT, Sharma R, Lim JL, Haider L, Frischer JM, Drexhage J, Mahad D, Bradl M, van Horssen J, Lassmann H (2012). NADPH oxidase expression in active multiple sclerosis lesions in relation to oxidative tissue damage and mitochondrial injury. Brain.

[182] Campbell GR, Kraytsberg Y, Krishnan KJ, Ohno N, Ziabreva I, Reeve A, Trapp BD, Newcombe J, Reynolds R, Lassmann H, Khrapko K, Turnbull DM, Mahad DJ (2012). Clonally expanded mitochondrial DNA deletions within the choroid plexus in multiple sclerosis. Acta Neuropathol.

[183] Pandit A, Vadnal J, Houston S, Freeman E, McDonough J (2009). Impaired regulation of electron transport chain subunit genes by nuclear respiratory factor 2 in multiple sclerosis. J Neurol Sci.

[184] Pache M, Kaiser HJ, Akhalbedashvili N, Lienert C, Dubler B, Kappos L, Flammer J (2003). Extraocular blood flow and endothelin-1 plasma levels in patients with multiple sclerosis. Eur Neurol.

[185] Speciale L, Sarasella M, Ruzzante S, Caputo D, Mancuso R, Calvo MG, Guerini FR, Ferrante P (2000). Endothelin and nitric oxide levels in cerebrospinal fluid of patients with multiple sclerosis. J Neurovirol.

[186] Matsushita T, Isobe N, Kawajiri M, Mogi M, Tsukuda K, Horiuchi M, Ohyagi Y, Kira J (2010). CSF angiotensin II and angiotensin-converting enzyme levels in anti-aquaporin-4 autoimmunity. J Neurol Sci.

[187] Constantinescu CS, Goodman DB, Grossman RI, Mannon LJ, Cohen JA (1997). Serum angiotensin-converting enzyme in multiple sclerosis. Arch Neurol.

[188] Platten M, Youssef S, Hur EM, Ho PP, Han MH, Lanz TV, Phillips LK, Goldstein MJ, Bhat R, Raine CS, Sobel RA, Steinman L (2009). Blocking angiotensin-converting enzyme induces potent regulatory T cells and modulates TH1- and TH17-mediated autoimmunity. Proc Natl Acad Sci U S A.

[189] Griffioen AW, Molema G (2000). Angiogenesis: potentials for pharmacologic intervention in the treatment of cancer, cardiovascular diseases, and chronic inflammation. Pharmacol Rev.

[190] Krum JM, Khaibullina A (2003). Inhibition of endogenous VEGF impende revascularization and astroglial proliferation: roles for VEGF in brain repair. Exp Neurol.

[191] Bouérat L, Fensholdt J, Liang X, Havez S, Nielsen SF, Hansen JR, Bolvig S, Andersson C (2005). Indolin-2-ones with high in vivo efficacy in a model for multiple sclerosis. J Med Chem.

[192] Mohty M, Brissot E, Savani BN, Gaugler B (2013). Effects of bortezomib on the immune system: a focus on immune regulation. Biol Blood Marrow Transplant.

[193] Dredge K, Marriott JB, Dalgleish AG (2002). Immunological effects of thalidomide and its chemical and functional analogs. Crit Rev Immunol.

[194] Sastry PS (1999). Inhibition of TNF-alpha synthesis with thalidomide for prevention of acute exacerbations and altering the natural history of multiple sclerosis. Med Hypotheses.

[195] Sharief MK, Thompson EJ (1992). In vivo relationship of tumor necrosis factor-alpha to blood–brain barrier damage in patients with active multiple sclerosis. J Neuroimmunol.

[196] Faure M, Lejeune JP, Gaucherand M, Thivolet J (1981). PMN leukocytes chemotaxis: inhibition by thalidomide. Pathol Biol (Paris).

[197] McHugh SM, Rifkin IR, Deighton J, Wilson AB, Lachmann PJ, Lockwood CM, Ewan PW (1995). The immunosuppressive drug thalidomide induces T helper cell type 2 (Th2) and concomitantly inhibits Th1 cytokine production in mitogen- and antigen-stimulated human peripheral blood mononuclear cell cultures. Clin Exp Immunol.

[198] Haslett PA, Corral LG, Albert M, Kaplan G (1998). Thalidomide costimulates primary human T lymphocytes, preferentially inducing proliferation, cytokine production, and cytotoxic responses in the CD8+ subset. J Exp Med.

[199] Ryu JK, Jantaratnotai N, McLarnon JG (2009). Thalidomide inhibition of vascular remodeling and inflammatory reactivity in the quinolinic acid-injected rat striatum. Neuroscience.

[200] Contino-Pépin C, Parat A, Périno S, Lenoir C, Vidal M, Galons H, Karlik S, Pucci B (2009). Preliminary biological evaluations of new thalidomide analogues for multiple sclerosis application. Bioorg Med Chem Lett.

[201] MacLean HJ, Freedman MS (2001). Immunologic therapy for relapsing – remitting multiple sclerosis. Curr Neurol Neurosci Rep.

[202] Folkman J, Langer R, Linhardt RJ, Haudenschild C, Taylor S (1983). Angiogenesis inhibition and tumor regression caused by heparin or a heparin fragment in the presence of cortisone. Science.

[203] Nauck M, Karakiulakis G, Perruchoud AP, Papakonstantinou E, Roth M (1998). Corticosteroids inhibit the expression of the vascular endothelial growth factor gene in human vascular smooth muscle cells. Eur J Pharmacol.

[204] Jablonska J, Leschner S, Westphal K, Lienenklaus S, Weiss S (2010). Neutrophils responsive to endogenous IFN-beta regulate tumor angiogenesis and growth in a mouse tumor model. J Clin Invest.

[205] Taylor KL, Leaman DW, Grane R, Mechti N, Borden EC, Lindner DJ (2008). Identification of interferon-beta-stimulated genes that inhibit angiogenesis in vitro. J Interferon Cytokine Res.

[206] Ewalt KL, Schimmel P (2002). Activation of angiogenic signaling pathways by two human tRNA synthetases. Biochemistry.

[207] Thamilarasan M, Hecker M, Goertsches RH, Paap BK, Schröder I, Koczan D, Thiesen HJ, Zettl UK (2013). Glatiramer acetate treatment effects on gene expression in monocytes of multiple sclerosis patients. J Neuroinflammation.

[208] Jin H, Su J, Garmy-Susini B, Kleeman J, Varner J (2006). Integrin alpha4beta1 promotes monocyte trafficking and angiogenesis in tumors. Cancer Res.

[209] Podar K, Zimmerhackl A, Fulciniti M, Tonon G, Hainz U, Tai YT, Vallet S, Halama N, Jäger D, Olson DL, Sattler M, Chauhan D, Anderson KC (2011). The selective adhesion molecule inhibitor Natalizumab decreases multiple myeloma cell growth in the bone marrow microenvironment: therapeutic implications. Br J Haematol.

[210] Foster CA, Mechtcheriakova D, Storch MK, Balatoni B, Howard LM, Bornancin F, Wlachos A, Sobanov J, Kinnunen A, Baumruker T (2009). FTY720 rescue therapy in the dark agouti rat model of experimental autoimmune encephalomyelitis: expression of central nervous system genes and reversal of blood–brain-barrier damage. Brain Pathol.

[211] Miron VE, Schubart A, Antel JP (2008). Central nervous system-directed effects of FTY720 (fingolimod). J Neurol Sci.

[212] Mousseau Y, Mollard S, Richard L, Nizou A, Faucher-Durand K, Cook-Moreau J, Qiu H, Baaj Y, Funalot B, Fourcade L, Sturtz FG (2012). Fingolimod inhibits PDGF-B-induced migration of vascular smooth muscle cell by down-regulating the S1PR1/S1PR3 pathway. Biochimie.

[213] Pulaski HL, Spahlinger G, Silva IA, McLean K, Kueck AS, Reynolds RK, Coukos G, Conejo-Garcia JR, Buckanovich RJ (2009). Identifying alemtuzumab as an anti-myeloid cell antiangiogenic therapy for the treatment of ovarian cancer. J Transl Med.

[214] Arnold P, Mojumder D, Detoledo J, Lucius R, Wilms H (2014). Pathophysiological processes in multiple sclerosis: focus on nuclear factor erythroid-2-related factor 2 and emerging pathways. Clin Pharmacol.

[215] García-Caballero M, Marí-Beffa M, Medina MÁ, Quesada AR (2011). Dimethylfumarate inhibits angiogenesis in vitro and in vivo: a possible role for its antipsoriatic effect?. J Invest Dermatol.

[216] Billington DC (1991). Angiogenesis and its inhibition: potential new therapies in oncology and non-neoplastic diseases. Drug Des Discov.

[217] Patten SG, Adamcic U, Lacombe K, Minhas K, Skowronski K, Coomber BL (2010). VEGFR2 heterogeneity and response to anti-angiogenic low dose metronomic cyclophosphamide treatment. BMC Cancer.

[218] Gora-Tybor J, Blonski JZ, Robak T (2002). Cladribine decreases the level of angiogenic factors in patients with chronic lymphocytic leukemia. Neoplasma.

[219] Wu X, Zhong H, Song J, Damoiseaux R, Yang Z, Lin S (2006). Mycophenolic acid is a potent inhibitor of angiogenesis. Arterioscler Thromb Vasc Biol.

[220] Brundula V, Rewcastle NB, Metz LM, Bernard CC, Yong VW (2002). Targeting leukocyte MMPs and transmigration Minocycline as a potential therapy for multiple sclerosis. Brain.

[221] Weingart JD, Sipos EP, Brem H (1995). The role of minocycline in the treatment of intracranial 9 L glioma. J Neurosurg.

[222] Tamargo RJ, Bok RA, Brem H (1991). Angiogenesis inhibition by minocycline. Cancer Res.

[223] Yao JS, Chen Y, Zhai W, Xu K, Young WL, Yang GY (2004). Minocycline exerts multiple inhibitory effects on vascular endothelial growth factor-induced smooth muscle cell migration: the role of ERK1/2, PI3K, and matrix metalloproteinases. Circ Res.

[224] Garrido-Mesa N, Zarzuelo A, Gálvez J (2013). Minocycline: far beyond an antibiotic. Br J Pharmacol.

[225] Hauser SL, Oksenberg JR (2006). The neurobiology of multiple sclerosis: genes, inflammation, and neurodegeneration. Neuron.

[226] Rovaris M, Confavreux C, Furlan R, Kappos L, Comi G, Filippi M (2006). Secondary progressive multiple sclerosis: current knowledge and future challenges. Lancet Neurol.

[227] Herrán E, Pérez-González R, Igartua M, Pedraz JL, Carro E, Hernández RM (2013). VEGF-releasing biodegradable nanospheres administered by craniotomy: a novel therapeutic approach in the APP/Ps1 mouse model of Alzheimer's disease. J Control Release.

[228] Hwang DH, Lee HJ, Park IH, Seok JI, Kim BG, Joo IS, Kim SU (2009). Intrathecal transplantation of human neural stem cells overexpressing VEGF provide behavioral improvement, disease onset delay and survival extension in transgenic ALS mice. Gene Ther.

[229] Tian YY, Tang CJ, Wang JN, Feng Y, Chen XW, Wang L, Qiao X, Sun SG (2007). Favorable effects of VEGF gene transfer on a rat model of Parkinson’s disease using adeno-associated viral vectors. Neurosci Lett.

[230] Azzouz M, Ralph GS, Storkebaum E, Walmsley LE, Mitrophanous KA, Kingsman SM, Carmeliet P, Mazarakis ND (2004). VEGF delivery with retrogradely transported lentivector prolongs survival in a mouse ALS model. Nature.

[231] Sun Y, Jin K, Xie L, Childs J, Mao XO, Logvinova A, Greenberg DA (2003). VEGF-induced neuroprotection, neurogenesis, and angiogenesis after focal cerebral ischemia. J Clin Invest.

[232] Tang Z, Yin JX, Han P, Gan Y, Coons SW, Wang C, Maalouf M, Shi J (2013). Pertussis toxin attenuates experimental autoimmune encephalomyelitis by upregulating neuronal vascular endothelial growth factor. Neuroreport.

[233] Nowak DG, Woolard J, Amin EM, Konopatskaya O, Saleem MA, Churchill AJ, Ladomery MR, Harper SJ, Bates DO (2008). Expression of pro- and anti-angiogenic isoforms of VEGF is differentially regulated by splicing and growth factors. J Cell Sci.

[234] Magnussen AL, Rennel ES, Hua J, Bevan HS, Beazley Long N, Lehrling C, Gammons M, Floege J, Harper SJ, Agostini HT, Bates DO, Churchill AJ (2010). VEGF-A165b is cytoprotective and antiangiogenic in the retina. Invest Ophthalmol Vis Sci.

[235] MacMillan CJ, Doucette CD, Warford J, Furlong SJ, Hoskin DW, Easton AS (2014). Murine experimental autoimmune encephalomyelitis is diminished by treatment with the angiogenesis inhibitors B20-4.1.1 and angiostatin (K1-3). PLoS One.

[236] Lopez-Ramirez MA, Wu D, Pryce G, Simpson JE, Reijerkerk A, King-Robson J, Kay O, de Vries HE, Hirst MC, Sharrack B, Baker D, Male DK, Michael GJ, Romero IA (2014). MicroRNA-155 negatively affects blood–brain barrier function during neuroinflammation. FASEB J.

[237] Munger KL, Zhang SM, O'Reilly E, Hernán MA, Olek MJ, Willett WC, Ascherio A (2004). Vitamin D intake and incidence of multiple sclerosis. Neurology.

[238] Grundmann M, Haidar M, Placzko S, Niendorf R, Darashchonak N, Hubel CA, von Versen-Höynck F (2012). Vitamin D improves the angiogenic properties of endothelial progenitor cells. Am J Physiol Cell Physiol.

[239] Gazzerro P, Proto MC, Gangemi G, Malfitano AM, Ciaglia E, Pisanti S, Santoro A, Laezza C, Bifulco M (2012). Pharmacological actions of statins: a critical appraisal in the management of cancer. Pharmacol Rev.

[240] Wang B, Sun L, Tian Y, Li Z, Wei H, Wang D, Yang Z, Chen J, Zhang J, Jiang R (2012). Effects of atorvastatin in the regulation of circulating EPCs and angiogenesis in traumatic brain injury in rats. J Neurol Sci.

[241] Birnbaum G, Cree B, Altafullah I, Zinser M, Reder AT (2008). Combining beta interferon and atorvastatin may increase disease activity in multiple sclerosis. Neurology.

[242] Kamm CP, El-Koussy M, Humpert S, Findling O, Burren Y, Schwegler G, Donati F, Müller M, Müller F, Slotboom J, Kappos L, Naegelin Y, Mattle HP, SWABIMS Study Group (2014). Atorvastatin added to interferon beta for relapsing multiple sclerosis: 12-month treatment extension of the randomized multicenter SWABIMS Trial. PLoS One.

[243] Stüve O, Youssef S, Weber MS, Nessler S, von Büdingen HC, Hemmer B, Prod'homme T, Sobel RA, Steinman L, Zamvil SS (2006). Immunomodulatory synergy by combination of atorvastatin and glatiramer acetate in treatment of CNS autoimmunity. J Clin Invest.

[244] Sorensen PS, Lycke J, Erälinna JP, Edland A, Wu X, Frederiksen JL, Oturai A, Malmeström C, Stenager E, Sellebjerg F, Sondergaard HB, SIMCOMBIN study investigators (2011). Simvastatin as add-on therapy to interferon β-1a for relapsing-remitting multiple sclerosis (SIMCOMBIN study): a placebo-controlled randomised phase 4 trial. Lancet Neurol.

[245] Dell'Acqua ML, Lorenzini L, D'Intino G, Sivilia S, Pasqualetti P, Panetta V, Paradisi M, Filippi MM, Baiguera C, Pizzi M, Giardino L, Rossini PM, Calzà L (2012). Functional and molecular evidence of myelin- and neuroprotection by thyroid hormone administration in experimental allergic encephalomyelitis. Neuropathol Appl Neurobiol.

[246] Bartels C, Späte K, Krampe H, Ehrenreich H (2008). Recombinant human Erythropoietin: novel strategies for neuroprotective/neuro-regenerative treatment of multiple sclerosis. Ther Adv Neurol Disord.

[247] Créange A, Lefaucheur JP, Balleyguier MO, Galactéros F (2013). Iron depletion induced by bloodletting and followed by rhEPO administration as a therapeutic strategy in progressive multiple sclerosis: a pilot, open-label study with neurophysiological measurements. Neurophysiol Clin.

[248] Ehrenreich H, Fischer B, Norra C, Schellenberger F, Stender N, Stiefel M, Sirén AL, Paulus W, Nave KA, Gold R, Bartels C (2007). Exploring recombinant human erythropoietin in chronic progressive multiple sclerosis. Brain.

[249] Najmi Varzaneh F, Najmi Varzaneh F, Azimi AR, Rezaei N, Sahraian MA (2014). Efficacy of combination therapy with erythropoietin and methylprednisolone in clinical recovery of severe relapse in multiple sclerosis. Acta Neurol Belg.

[250] Balashov KE, Aung LL, Dhib-Jalbut S, Keller IA (2011). Acute multiple sclerosis lesion: conversion of restricted diffusion due to vasogenic edema. J Neuroimaging.

[251] Tievsky AL, Ptak T, Farkas J (1999). Investigation of apparent diffusion coefficient and diffusion tensor anisotrophy in acute and chronic multiple sclerosis lesions. AJNR Am J Neuroradiol.

[252] Zlokovic BV (2008). The blood–brain barrier in health and chronic neurodegenerative disorders. Neuron.

[253] Muramatsu R, Takahashi C, Miyake S, Fujimura H, Mochizuki H, Yamashita T (2012). Angiogenesis induced by CNS inflammation promotes neuronal remodeling through vessel-derived prostacyclin. Nat Med.

[254] Li L, Welser JV, Dore-Duffy P, del Zoppo GJ, Lamanna JC, Milner R (2010). In the hypoxic central nervous system, endothelial cell proliferation is followed by astrocyte activation, proliferation, and increased expression of the alpha 6 beta 4 integrin and dystroglycan. Glia.

[255] Palavra F, Marado D, Mascarenhas-Melo F, Sereno J, Teixeira-Lemos E, Nunes CC, Gonçalves G, Teixeira F, Reis F (2013). New markers of early cardiovascular risk in multiple sclerosis patients: oxidized-LDL correlates with clinical staging. Dis Markers.

[256] Chaitanya GV, Omura S, Sato F, Martinez NE, Minagar A, Ramanathan M, Guttman BW, Zivadinov R, Tsunoda I, Alexander JS (2013). Inflammation induces neuro-lymphatic protein expression in multiple sclerosis brain neurovasculature. J Neuroinflammation.

[257] Sarchielli P, Di Filippo M, Ercolani MV, Chiasserini D, Mattioni A, Bonucci M, Tenaglia S, Eusebi P, Calabresi P (2008). Fibroblast growth factor-2 levels are elevated in the cerebrospinal fluid of multiple sclerosis patients. Neurosci Lett.

[258] Rieckmann P, Albrecht M, Ehrenreich H, Weber T, Michel U (1995). Semi-quantitative analysis of cytokine gene expression in blood and cerebrospinal fluid cells by reverse transcriptase polymerase chain reaction. Res Exp Med (Berl).

[259] Giovannoni G (1998). Cerebrospinal fluid and serum nitric oxide metabolites in patients with multiple sclerosis. Mult Scler.

[260] Sarchielli P, Orlacchio A, Vicinanza F, Pelliccioli GP, Tognoloni M, Saccardi C, Gallai V (1997). Cytokine secretion and nitric oxide production by mononuclear cells of patients with multiple sclerosis. J Neuroimmunol.

[261] Maimone D, Gregory S, Arnason BG, Reder AT (1991). Cytokine levels in the cerebrospinal fluid and serum of patients with multiple sclerosis. J Neuroimmunol.

[262] Hauser SL, Doolittle TH, Lincoln R, Brown RH, Dinarello CA (1990). Cytokine accumulations in CSF of multiple sclerosis patients: frequent detection of interleukin-1 and tumor necrosis factor but not interleukin-6. Neurology.

[263] Sharief MK, Hentges R (1991). Association between tumor necrosis factor-alpha and disease progression in patients with multiple sclerosis. N Engl J Med.

[264] Kahl KG, Kruse N, Toyka KV, Rieckmann P (2002). Serial analysis of cytokine mRNA profiles in whole blood samples from patients with early multiple sclerosis. J Neurol Sci.

[265] Link J (1994). Interferon-gamma, interleukin-4 and transforming growth factor-beta mRNA expression in multiple sclerosis and myasthenia gravis. Acta Neurol Scand Suppl.

[266] Rieckmann P, Albrecht M, Kitze B, Weber T, Tumani H, Broocks A, Lüer W, Helwig A, Poser S (1995). Tumor necrosis factor-alpha messenger RNA expression in patients with relapsing-remitting multiple sclerosis is associated with disease activity. Ann Neurol.

[267] Nicoletti F, Di Marco R, Patti F, Reggio E, Nicoletti A, Zaccone P, Stivala F, Meroni PL, Reggio A (1998). Blood levels of transforming growth factor-beta 1 (TGF-beta1) are elevated in both relapsing remitting and chronic progressive multiple sclerosis (MS) patients and are further augmented by treatment with interferon-beta 1b (IFN-beta1b). Clin Exp Immunol.

[268] Rollnik JD, Sindern E, Schweppe C, Malin JP (1997). Biologically active TGF-beta 1 is increased in cerebrospinal fluid while it is reduced in serum in multiple sclerosis patients. Acta Neurol Scand.

[269] Hollifield RD, Harbige LS, Pham-Dinh D, Sharief MK (2003). Evidence for cytokine dysregulation in multiple sclerosis: peripheral blood mononuclear cell production of pro-inflammatory and anti-inflammatory cytokines during relapse and remission. Autoimmunity.

[270] Hohnoki K, Inoue A, Koh CS (1998). Elevated serum levels of IFN-gamma, IL-4 and TNF-alpha/unelevated serum levels of IL-10 in patients with demyelinating diseases during the acute stage. J Neuroimmunol.

[271] Avolio C, Ruggieri M, Giuliani F, Liuzzi GM, Leante R, Riccio P, Livrea P, Trojano M (2003). Serum MMP-2 and MMP-9 are elevated in different multiple sclerosis subtypes. J Neuroimmunol.

[272] Fainardi E, Castellazzi M, Tamborino C, Trentini A, Manfrinato MC, Baldi E, Tola MR, Dallocchio F, Granieri E, Bellini T (2009). Potential relevance of cerebrospinal fluid and serum levels and intrathecal synthesis of active matrix metalloproteinase-2 (MMP-2) as markers of disease remission in patients with multiple sclerosis. Mult Scler.

[273] Bar-Or A, Nuttall RK, Duddy M, Alter A, Kim HJ, Ifergan I, Pennington CJ, Bourgoin P, Edwards DR, Yong VW (2003). Analyses of all matrix metalloproteinase members in leukocytes emphasize monocytes as major inflammatory mediators in multiple sclerosis. Brain.

[274] Lee MA, Palace J, Stabler G, Ford J, Gearing A, Miller K (1999). Serum gelatinase B, TIMP-1 and TIMP-2 levels in multiple sclerosis. A longitudinal clinical and MRI study. Brain.

[275] Fainardi E, Castellazzi M, Bellini T, Manfrinato MC, Baldi E, Casetta I, Paolino E, Granieri E, Dallocchio F (2006). Cerebrospinal fluid and serum levels and intrathecal production of active matrix metalloproteinase-9 (MMP-9) as markers of disease activity in patients with multiple sclerosis. Mult Scler.

[276] Leppert D, Ford J, Stabler G, Grygar C, Lienert C, Huber S, Miller KM, Hauser SL, Kappos L (1998). Matrix metalloproteinase-9 (gelatinase B) is selectively elevated in CSF during relapses and stable phases of multiple sclerosis. Brain.

[277] Boz C, Ozmenoglu M, Velioglu S, Kilinc K, Orem A, Alioglu Z, Altunayoglu V (2006). Matrix metalloproteinase-9 (MMP-9) and tissue inhibitor of matrix metalloproteinase (TIMP-1) in patients with relapsing-remitting multiple sclerosis treated with interferon beta. Clin Neurol Neurosurg.

[278] Bomprezzi R, Ringnér M, Kim S, Bittner ML, Khan J, Chen Y, Elkahloun A, Yu A, Bielekova B, Meltzer PS, Martin R, McFarland HF, Trent JM (2003). Gene expression profile in multiple sclerosis patients and healthy controls: identifying pathways relevant to disease. Hum Mol Genet.

[279] Duan H, Luo Y, Hao H, Feng L, Zhang Y, Lu D, Xing S, Feng J, Yang D, Song L, Yan X (2013). Soluble CD146 in cerebrospinal fluid of active multiple sclerosis. Neuroscience.

